# Social Support as a Stress Buffer or Stress Amplifier and the Moderating Role of Implicit Motives: Protocol for a Randomized Study

**DOI:** 10.2196/39509

**Published:** 2022-08-09

**Authors:** Alisa Haufler, Beate Ditzen, Julia Schüler

**Affiliations:** 1 Department of Sport Science University of Konstanz Constance Germany; 2 Institute for Medical Psychology Heidelberg University Hospital Heidelberg Germany; 3 Heidelberg University Heidelberg Germany

**Keywords:** stress, Trier Social Stress Test, social support, social motives, cortisol, reproductive hormones

## Abstract

**Background:**

Previous research shows that providing social support in socioevaluative stress situations reduces participants’ stress responses. This stress-buffer effect, however, does not hold for everybody, and some studies even found a stress-amplifying effect of social support. Motive disposition research suggests that social motives (affiliation and power) lead to differential and sometimes even opposing affective and physiological responses to interpersonal interaction processes. We here integrate both lines of research and hypothesize that participants with strong affiliation motives benefit, while participants with strong power motives do not benefit from social support in terms of psychobiological responses to a given stressor. Further, participants with strong affiliation and power motives are expected to respond to social support with the arousal of motive-specific affects and reproductive hormone responses (affiliation: progesterone; power: estradiol and testosterone). In addition, we test sex differences in the response to social support and in the strengths of social motives.

**Objective:**

The main objective of this study is to test whether social motives and participants’ sex moderate the effects of social support in stressful situations.

**Methods:**

We aim to collect data from 308 participants recruited at our local university. Participants’ social motives are assessed using a standardized measure in motive research (Picture Story Exercise). Then, the Trier Social Stress Test for Groups (TSST-G) is used to experimentally induce psychosocial stress. One group of participants receives social support from an associate of the experimenter, while the control group does not receive social support. Stress responses will be assessed by a modified version of the state anxiety scale of the State–Trait Anxiety Inventory and by physiological indicators of stress (cortisol and α-amylase from saliva samples) at 7 measurement points. Reproductive hormones will be analyzed in 4 of these 7 saliva samples. Heart rate and heart rate variability will be assessed continuously. We will additionally measure participants’ performance in an interview (part of the TSST-G) using a self-developed categorization system.

**Results:**

The Ethics Committee of the University of Constance approved the application to conduct the study on December 18, 2018. Furthermore, the study was retrospectively registered in the German Clinical Trials Register (DKRS; ID: DRKS00028503) on March 09, 2022. The start of the experiment was planned for the beginning of 2019, but was postponed to June 2021 due to COVID-19. Publication of the first results is planned for spring 2023.

**Conclusions:**

Our theory-driven integration of social motives in social support research and the precise analysis of sex differences might disentangle inconsistent findings in TSST research. The more faceted view on individual differences has direct implications for applied contexts as it provides a framework for tailored conceptualizations of social support programs.

**Trial Registration:**

German Clinical Trials Register DRKS00028503; https://tinyurl.com/5a87x4da

**International Registered Report Identifier (IRRID):**

PRR1-10.2196/39509

## Introduction

### Stress and Social Support

There is an extended body of research outlining that stress affects basically every physiological system [1,2] and significantly impairs subjective well-being [3,4]. Therefore, it is unsurprising that the World Health Organization anticipates stress-related illness to progress to the second leading cause of disease in the coming decades [5]. Hence, it is essential to better understand the complexity of the concept of stress to be able to develop effective interventions. In the last decades, a great deal of research has shown that social support, defined as “social interactions or relationships that provide individuals with actual assistance or with a feeling of attachment to a person or group that is perceived as loving or caring” [6], can improve health [7-10]. One of the leading explanations for this phenomenon is that social support has a stress-buffering effect [11,12] and can thus counteract the negative consequences of stress. For example, social support leads to lower mortality rates [13,14], and better recovery from surgery [15] and sport injuries [16]. Yet, interestingly social support does not work as a stress buffer for everyone [8]. We assume that social support is perceived differently by individuals and investigate social motives (affiliation and power motive) [17] as moderators. They influence the perception of interpersonal relationships and should therefore also explain responses to social support.

### Implicit Motives

Implicit motives are preferences for certain kinds of incentives and disincentives, which modulate reward experiences [17-21]. Being relatively stable across time (such as personality traits), they drive, orient, and select behaviors for summaries [22]. Motive research has focused on the 3 domains of affiliation, power, and achievement motives, of which, we consider only social motives in our study. 

Individuals with a strong affiliation motive derive pleasure from affiliative experiences [17,23]. They have the desire for warm and friendly interpersonal relations [24], aim to feel socially related, want to experience reciprocal care and concern for important others [17,25], and emotionally suffer from discord, rejection, and loneliness [17,25,26]. Situations in which these needs can be satisfied lead to an affiliation motive–specific affect, such as joy, and feeling socially related [27].

Individuals with a strong power motive have the desire to have an impact on others and influence others (in socially desirable and undesirable ways) in order to feel superior to others and gain or maintain reputation and prestige [28,29]. Simultaneously, they aim to avoid defeat, other’s dominance, and feelings of inferiority [30]. In brief, they have “the capacity to derive pleasure from having physical, mental, or emotional impact on other individuals or groups of individuals and to experience the impact of others on themselves as aversive” [17]. The lack of opportunities in which others can be impacted or, even worse, situations signaling one’s inferiority function as stressors and lead to a power motive–specific affect (eg, feeling inferior and experiencing limited control) and impaired well-being [31,32]. 

Social motives are also associated with specific hormones [33,34]. Being inferior, for example, in a contest situation, has been associated with a decrease in testosterone in men with strong power motives [35]. For high power–motivated women, motive frustration leads to a decrease in estradiol [35]. Arousal of the affiliation motive is accompanied by an increase in progesterone for both sexes [33,36,37]. Furthermore, social motives in relationship with stress have been associated with various parameters of health, including blood pressure and the immune system [20], medication use and somatic symptoms [38], or job burnout and physical symptoms [39]. In summary, previous research has confirmed that affiliation and power motives lead to differential emotional, behavioral, and physiological responses to social cues. 

### Social Support and Social Motives

Based on the evidence that both social support and social motives modulate the stress response, we aim to investigate to what extent the interplay of these 2 factors can contribute to further enlightenment of the stress-buffer effect. Social support situations are highly ambiguous, leaving wide room for interpretation about, for example, one’s position in the social context, the intentions of the social support provider, and the quality of social relationships. By this, they are prototypes of social interaction processes, which are full of incentives or disincentives for social motives. They can, however, be perceived very differently by individuals with strong affiliation in contrast to power motives and therefore elicit different physiological and psychological responses. Thus, social support might signal a positive and warm relationship for individuals with a strong affiliation motive, but trigger feelings of weakness and inferiority in individuals with a strong power motive. In summary, we assume that social motives influence the perception of social support provided by others and function as a stress buffer in affiliation-motivated individuals and as a stress amplifier in power-motivated individuals.

### Social Support, Sex, and Gender

Other moderators that are discussed to influence participants’ responses to social support are sex and gender [8,40,41]. Women benefit stronger in terms of well-being from being socially supported than men [42,43], even though some studies found opposite effects [44]. Thus, the empirical evidence on whether and how women and men differ in their responses to social support is inconsistent. 

Sex differences have also been found in social motive research. Since the arousal of the affiliation and power motives in a specific situation is accompanied by the release of female reproductive hormones (estradiol and progesterone) and a male reproductive hormone (testosterone), it is assumed that this sex specificity should also be reflected in corresponding motive differences. Women are expected to show higher scores in affiliation motives, and men are assumed to have higher power motives. This was clearly empirically supported for the affiliation motive [45,46], whereas for the power motive, the result pattern is less clear [45,47]. These motives are assumed to correlate with a concept on a broader level of abstraction, that is, gender role self-concept (GRSC) [48]. The individual GRSC is defined as describing oneself with agentic traits like confident or assertive (masculine GRSC) versus with communal traits like empathic or cooperative (feminine GRSC). We assume that the inconsistent findings reported above may be due to shared variance among sex, GRSC, and motives. We aim to identify the specific influences of sex, GRSC, and social motives on the stress response to social support by considering them simultaneously and disentangling them in our statistical analyses.

### Planned Research

The main objective of this study is to test whether social motives and participants’ sex moderate the effects of social support in stressful situations. We use the Trier Social Stress Test for Groups (TSST-G) [49] that is based on the Trier Social Stress Test (TSST) [50], which is an established stress-induction paradigm triggering strong psychobiological stress responses [51]. Schultheiss et al [52] found that the TSST elicited differently strong cortisol responses for individuals with weak and strong implicit achievement motives, which supports our assumption that the TSST might be a potentially suitable paradigm that reveals motive differences. Wiemers et al [53] concluded from their study that the TSST has a specific arousal effect for the implicit power motive.

This study varies from classic TSST studies in the following aspects. While TSST studies usually focus on the detection of the stress hormone cortisol [54,55], we add the analysis of reproductive hormones (progesterone, estradiol, and testosterone), which will allow us to examine the arousal of motives in social support situations. We will further extend the TSST paradigm by analyzing participants’ responses in an interview (part of the TSST) to obtain an indicator for speech performance. While in the classical TSST paradigm, it is only announced that speech will be recorded (as an additional stressor), we here will actually record speech and apply a simple evaluation system to assess speech performance as a variable that we assume depends on stress.

**Figure 1:**
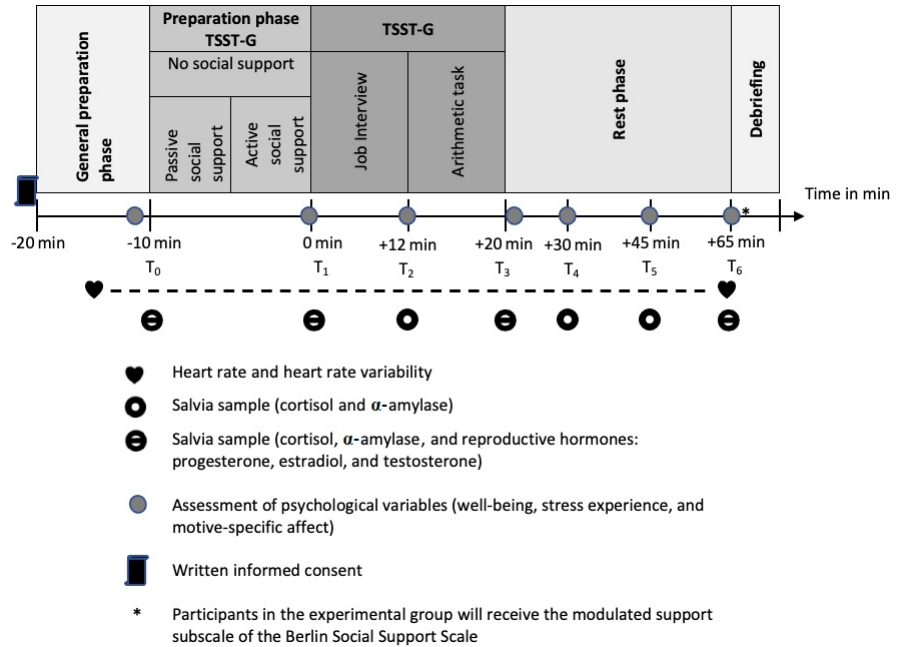
Trier Social Stress Test for groups (TSST-G): Phases of the procedure.

Except for these variations, we will adhere to the procedure of the TSST-G [49]. As in previous studies analyzing social support [40,41,56], the experimental group will receive social support during the TSST preparation phase. The control group will also be exposed to stress but will not receive social support. To test the study hypotheses (see below), self-reports (well-being, perceived stress, and motive-specific affect) and biological parameters (heart rate, heart rate variability, cortisol, and reproductive hormones) will be collected (Figure 1).

### Objectives

We will test the below hypotheses to investigate the role of social motives in the social support and stress relationship.

#### Hypothesis 1: Effect of Stress Induction Hypothesis

Participants in both groups (social support vs no social support) show an increase in stress responses comparable to that in previous studies. We expect a rise in the physiological parameter cortisol by at least 1.5 nmol/mL (Miller criterion) [57].

#### Hypothesis 2: Social Support × Social Motive Hypothesis

The affiliation motive moderates the effects of social support on stress responses. A higher affiliation motive of participants is associated with greater benefit from social support, that is, they will express lower psychobiological stress responses (better self-reported well-being, less perceived stress, lower heart rate, higher heart rate variability, and lower levels of cortisol and α-amylase). While the affiliation motive is expected to function as a stress buffer, the power motive is assumed to function as a stress amplifier. A higher power motive of participants is associated with greater negative impact from social support, that is, they will report lower well-being, more perceived stress, higher heart rate, lower heart rate variability, and higher levels of cortisol and α-amylase.

#### Hypothesis 3: Motive-Specific Arousal Hypothesis 

Participants with strong affiliation and power motives respond to social support with arousal of self-reported motive-specific affect (ie, affiliation: joy and feeling socially related; power: feeling weak and inferior) and with an increase in motive-specific reproductive hormone responses* *(affiliation: progesterone; power: estradiol and testosterone).

#### Hypothesis 4: Sex Difference Hypothesis

Women and men are hypothesized to differ in their social motives, with higher affiliation and lower power motives in women than in men. Women and men will specifically respond to social support with relative increases in estradiol and progesterone in women and testosterone in men.

#### Hypothesis 5: Speech Performance Hypothesis

Participants who receive social support show better presentation performance in TSST interviews than participants in the no social support group. This relationship is moderated by social motives. Participants in the social support group perform better when the affiliation motive is high and perform worse when the power motive is high.

#### Exploratory Hypothesis: GRSC

On an exploratory level, we plan to investigate the association of self-reported GRSC with social motives and their moderating role on whether individuals of either sex benefit from social support.

## Methods

### Study Registration

This study was retrospectively registered in the German Clinical Trials Register (DKRS) on March 09, 2022, under the following number: DRKS00028503. The trial was not prospectively registered because COVID-19 restrictions led to uncertainty about whether, when, and under what circumstances (eg, wearing a FFP2 mask) data collection could take place.

### Study Design

The study is based on a mixed within- and between-subject design. The within-subject factors are participants’ stress responses (self-reports and biological markers) across the steps of the TSST procedure (Figure 1). The between-subject factors are experimental groups (social support vs no social support), social motives, and participants’ sex. GRSC and other control variables will be assessed as controls and entered as covariates into the analysis models. Allocation will be based on a 1:1 ratio. There will be no cross-over into the experimental group.

The study will be conducted in the laboratory of sports psychology of the Department of Sport Science at the University of Konstanz, Germany. The analyses of hormones and α-amylase will be performed in the biochemical laboratory of the Institute for Medical Psychology in Heidelberg, Germany.

### Ethics Approval

The study was approved by the Institutional Review Board of the University of Konstanz on December 18, 2018 (35/2018). Further, the participants will receive a study information sheet and give their informed consent in the screening online survey (Multimedia Appendix 1) before the laboratory session. If they fulfill the inclusion criteria, they will be invited to the laboratory session. Here, the participants will again read the study information sheet and sign the informed consent (Multimedia Appendix 2). Participants can discontinue the study at any time without giving any reason. They will still receive their payment.

To ensure confidentiality**,** personal data (email addresses) of potential and enrolled participants will be collected by the principal investigator and stored password-protected on the local university server. The information will not be shared.

### Blinding

Participants and all experimenters involved in data collection and processing (eg, motive coders), with the only exception of social support providers, will be blinded to the intervention after assignment.

### Sample Size and Recruitment

It is intended to recruit 154 participants (77 women). This sample size was calculated using a power analysis involving G*Power 3.1 [58], with an additional 20% added to compensate for possible dropouts.

The recruitment will be done by flyer distribution in the University of Konstanz, as well as an entry in an online platform where experiments are offered by the departments of psychology and linguistics.

### Eligibility Criteria

The following primary criteria must be fulfilled to participate in the study: (1) informed consent for all aspects of the study (agreement with video recording and hormone collection); (2) age at least 18 years; and (3) ability to speak German as the native language.

Participants who meet the following exclusion criteria will be excluded from study participation: (1) previous participation in stress experiments, as well as psychology and sports students from the 5th semester and (2) presence of physical or mental illness, nicotine consumption, drug use, BMI of 30 or more, and regular use of medication (including hormonal contraception), as these could influence the physiological stress response [59].

### Participant Adherence

Participants will receive €40 (about US $42) payment at the end of the laboratory session independent of whether or when participants decide to discontinue the study. To promote participant adherence with the appointment and study protocol, they will receive a reminder email after having filled in the web survey and 1 day before their laboratory appointment.

### Procedure

#### Web Survey Prior to the Laboratory Session

The participants complete an online questionnaire (Limesurvey) at home. Here, the eligibility criteria are checked, the implicit motives are assessed by using the Picture Story Exercise (PSE) [60], and GRSCs [61], as well as other control variables, are measured. The exercise and sports activity questionnaire [62] will be administered to test a related but different research question (Multimedia Appendix 1). Participants, who are eligible for the study, will be invited to the laboratory session via email. Participants confirm their agreement that they will be contacted by email and learn that email addresses will be deleted after the appointment is made.

#### Laboratory Session

Figure 1 shows the schematic procedure of data collection. It starts with a general preparation phase, where the baseline measurements of physiological parameters (hormones), control variables, and psychological variables (self-reports about well-being, stress experience, and motive-specific affect) take place. In the preparation phase for the TSST-G, the participants prepare for the task. The participants in the experimental group receive social support during this phase, while the participants in the control group do not receive any social support. Finally, there will be a 45-minute rest period during which repeated physiological and psychological questionnaires will be completed. A detailed procedure is provided in Multimedia Appendix 3.

#### General Preparation Phase

In each laboratory session, 3 participants will arrive between 5:00 and 5:15 PM outside the laboratory and will be led individually to their own preparation room (so that they cannot contact each other). First, they will be asked to read the study information sheet again and then will provide their written consent (Multimedia Appendix 2). Afterwards, the participants will generate their participant code via paper and pencil format, which ensures that the saliva samples, as well as other collected data, can be stored anonymously. Then, they will complete a short day-screening questionnaire (Multimedia Appendix 4) on a computer, which will assess control variables (eg, sports, medication intake, smoking, and caffeine and alcohol consumption). Afterward, they will be asked to wear a pulse belt that contains a Polar H10 sensor (Polar Electro). When putting on the belt, the experimenter will leave the room. After that, the participants will provide an initial saliva sample at measurement time T0 (−11 min) and will complete the first test battery, including different questionnaires, on a computer (hereafter referred to as psychological questionnaires).

#### Preparation Phase of the TSST-G Without Social Support

For the preparation phase, each participant sits in a separate room. Here, they receive written instructions for the upcoming interview (Multimedia Appendix 5). After they have had 10 minutes of preparation time for this task, they give a saliva sample (T1, 0 min) and complete the psychological questionnaire again. Subsequently, the participants are individually led to their places in front of the panel.

#### Preparation Phase of the TSST-G With Social Support

The preparation phase is identical to the described scenario for the participants without social support, with the exception that the experimenter introduces a female associate as a student assistant who can assist the participants if needed. The female associate provides passive social support for the first 5 minutes, ostensibly working on the computer. In the second 5 minutes of the preparation phase, the associate gives active social support and also notes the reactions of the participants (Multimedia Appendix 6). At the end of the 10-minute preparation period, the associate asks the participants for a saliva sample (T1, 0 min) and tells the participants to complete the psychological questionnaire again. Then, rooms are changed. All other instructions are the same as in the group without social support.

#### TSST-G

##### Psychosocial Stress Induction

Our procedure is based on the TSST-G developed by Von Dawans et al [49]. Each participant is required to present his or her interview individually in front of the panel for 3 minutes at a time. During this time, the participant is interrupted in a standardized manner by the panel (Multimedia Appendix 7). After that, they give a saliva sample (T2, +12 min) and complete the psychological questionnaire. Subsequently, each participant has to perform the arithmetic task 3 times for 30 seconds. When this task is finished, they give another saliva sample (T3, +20 min). During this whole procedure, the participants are recorded with a microphone and a camera. The experimenter then leads the participants individually from the TSST-G test room to the respective preparation rooms. Here, they again fill in the psychological questionnaire.

##### Rest Phase

During the rest period, 3 additional saliva samples are collected (T4, +30 min; T5, +45 min; T6, +65 min). Participants who have received social support will complete a social support scale at T6 (+65 min) (Multimedia Appendix 8).

##### Debriefing

Finally, the experimenter leads all subjects to the TSST-G testing room and provides a debriefing about the aim of the study (Multimedia Appendix 9). Questions are answered as needed. The participants receive their payment and are dismissed.

### Manipulation Check of Social Support

To check whether social support was received as such by the participants, a modified version of the received support subscale of the Berlin Social Support Scale [63] will be used (Multimedia Appendix 8; T6, +65 min). Item wording is adapted to the study context. Two items from the original scale are deleted because they refer to instrumental support, and the social support in this study rather refers to emotional and informal social support. The item “This student assistant was there for me when I needed her” is also counted as instrumental support according to Schulz and Schwarzer [63], but it can also be understood as emotional support and therefore remains included. Items are rated on a 4-point Likert scale ranging from 1 (not true) to 4 (exactly true). The original version of the received support scale has good internal consistency (α=.83). In addition, participants will be asked directly whether they received support from the student assistant and whether they found this support helpful.

### Test Battery of Psychological Questionnaires

Participants are asked to complete the psychological questionnaire a total of 7 times (Figure 1).

Well-being will be captured via 6 items (short version A) from the Multidimensional Well-Being Questionnaire (MDBF) [64]. The items start with “Right now I feel…” and will be continued with 1 of the following adjectives: good, bad, alertness, fatigue, relaxed, and restlessness. The participants will be able to rate them on a 5-level scale labelled from *not at all* to *very*. A slightly modified version of the trait anxiety scale of the State-Trait Anxiety Inventory [65] will be used to assess the momentary anxiety of the participants. A total of 6 adapted items are included, which can be answered on a 5-point scale, as follows: “How big do you think your fear is at the moment?”, “How much do you feel physically uncomfortable right now?”, “How strong is your need to leave the situation?”, “How tense is your feeling right now?”, “How much are you in control of the situation?”, and “How stressed do you feel?”. To our knowledge, no standardized motive-specific affect questionnaire exists so far. We therefore created an adjective list that is theoretically derived from early work by McClelland [19] and added adjectives that have been used in more recent research [66] (see the self-determination theory [67]). Participants indicated for 7 items how they feel right now by using a 7-point response scale (1, *not at all* to 7, *very much*). The items are “socially related,” “calm” (affiliation motive–specific affect; the item “relaxed” from the MDBF will also be used in the analysis for the affiliation motive), “strong,” “excited,” and “enthusiastic” (power motive), with “competent” and “self-determined” as additional items representing achievement and autonomy motive–specific affect, respectively (Multimedia Appendix 10). Construct validity of this motive-specific affect scale will be checked.

### Social Motives

Implicit social motives are measured using the PSE [60], which is the most frequently used measure to assess implicit motives. Key validity criteria are met, interrater reliability is good, and retest reliability is satisfactory [34,60]. For further discussion, refer to a previous report [21]. The PSE will be part of the online questionnaire prior to the laboratory session (for validity of the computer version of the PSE) [68]. Participants will be instructed that they will see 6 different pictures, and for each of them, they should write a fictional story with a beginning, middle, and end. The pictures will be presented for 15 seconds, and then, a text box will appear, where they can type their story. Questions that help participants to organize their stories will be presented above the pictures (eg, “What is happening right now?” and “Who are the characters?”). For each story, the participants will have 4 minutes. After 3 minutes 30 seconds, a small reminder will appear asking them to finish the story. After the 4 minutes have elapsed, the next picture will appear. The 6 pictures “couple by the river,” “nightclub scene,” “sorrow,” “beachcombers,” “NewPic32,” and “NewPic9” will be presented (Multimedia Appendix 1). As recommended previously [60], 2 experienced coders will score the stories for the power and affiliation motives according to Winter’s scoring manual [69] (interrater reliability [intraclass correlation coefficient] is expected to be between 0.80 and 0.90). Disagreements between coders will be resolved by discussion [60]. Motive scores will be corrected by word count. For further details about test administration and scoring procedure, see a previous report [60].

All participants will complete a short German version of the Bem Sex Role Inventory to screen for GRSC [61].

### Endocrine Measurements

Saliva samples will be collected for the recording of cortisol, α-amylase, and reproductive hormones. Approximately 10 mL of saliva will be dispensed through a straw into Salicaps (IBL International) (Multimedia Appendix 11). After the study, all saliva samples will be frozen and stored at −20°C. Hormone and enzyme levels will be analyzed at the stress biomarkers laboratory at the Institute of Medical Psychology, Heidelberg University Hospital.

#### Salivary Cortisol

The concentration of cortisol in saliva will be recorded in ng/mL. Seven saliva samples will be collected using Salicaps (IBL International) at measurement time points T0 (−11 min before the TSST-G), T1 (TSST-G onset), T2 (after the job interview), T3 (after the arithmetic task), and T4, T5, and T6 during the resting phase (+30 min, +45 min, and +65 min, respectively, after TSST-G onset). Cortisol will be determined with the Cortisol free in Saliva ELISA assay from Demeditec.

#### Salivary α-Amylase

We will record α-amylase in U/mL. The concentration is derived from the same 7 salvia samples as used for the cortisol analysis and will be determined by a kinetic colorimetric test. The reagents for this will be obtained from DiaSys Diagnosic Systems.

#### Reproductive Hormones

Reproductive hormones (testosterone, estradiol, and progesterone) will be recorded in pg/mL. Four saliva samples will be collected using Salicaps (IBL International) at the measurement time points T0 (−11 min before the TSST-G), T1 (TSST-G onset), T3 (after the arithmetic task), and T6 during rest (+65 min after TSST-G onset). Hormone concentrations will be determined by biochemical analysis in the laboratory. The following kits from IBL will be used for analysis: Testosterone Luminescence Immunoassay, 17 beta-Estradiol Saliva Luminescence Immunoassay, and Progesterone Luminescence Immunoassay.

### Autonomic Nervous System

Heart rate and heart rate variability will be measured with a Polar H10 sensor (Polar Electro UK Ltd). The sensor is placed in a pulse belt that the participants will wear around their chest. With the help of a Polar station and an iPad, the participants’ data are transmitted wirelessly and in real time.

### Speech Performance

The participants will be videotaped while they complete the tasks (interview and arithmetic task) in front of the panel. The video sequences showing the recording of the interview will be coded for speech performance using a self-developed coding system. This system includes the following 3 quality criteria: the information content, the presentation style, and the perceived competence of the participants. The assessment of the information content is based on a checklist for the evaluation of a presentation according to Ascheron [70]. The content is scored on the following 5 items: “structure/organization,” “comprehensibility of content,” “flow,” “information content,” and “message.” These items will be rated on a scale that ranges from 1 (*very good*) to 6 (*unsatisfactory*). A modified questionnaire of Ascheron [70] will be used to evaluate the presentation style. The 2 items intonation and English quality were left out because intonation overlaps with another item (emphasis) and English quality is irrelevant because the study will be conducted in German. The presentation style is rated on the basis of the following 5 items: “speed,” “intelligibility,” “emphasis,” “body language,” and “eye contact,” whereby we added the latter item to complement the construct in more detail. The items will be scored using a 6-point scale (1, *very good* to 6, *unsatisfactory*). Since there is no suitable measurement tool for the assessment of perceived competence in the literature, we determined 5 items that should enable a differentiated evaluation of this construct. The following items will be scored on a 6-point scale: “technical language/vocabulary,” “use of filler words,” “use of everyday language,” “interest,” and “persuasiveness.” Construct validity of this competence scale will be checked.

### Data Management

The questionnaire data will be downloaded from Limesurvey and stored on the university server. The psychological questionnaire from T2, which will be collected by paper and pencil format, as well as heart rate and heart rate variability will be stored in an Excel table by the study experimenter directly after the study. The video file will also be saved directly after the experiment, on a laptop of the sport psychology laboratory and a back-up server. The saliva samples will be sent to the biochemical laboratory of the Institute for Medical Psychology in Heidelberg, Germany. To guarantee the accuracy of the analyses, 10% of the cortisol samples and 20% of the samples for reproductive hormones will be double assessed. The signed consent forms of the participants will be collected in the sport psychology laboratory in Constance. Only the experimenter will have access to the data, which will be stored for 10 years on a server of the University.

The participants will generate their own code that allows to merge the data of the web surveys with the data obtained in the laboratory.

Saliva samples will be stored in the biochemical laboratory at Heidelberg University Hospital for at least 2 years after completion of the study and will then be discarded.

### Statistical Analysis

Statistical analysis will be performed using IBM SPSS Statistics, Version 28 (IBM Corp) for Windows (statistical analysis and graphs). We will perform an analysis of variance with repeated measures to determine if the TSST procedure results in significant increases in psychological and biological variables for all participants. We will calculate multiple linear regressions to examine interaction effects. In these regressions, we will first include the control variables, then the condition as a dummy variable, and finally the individual predictors and their interaction terms. To account for repeated measurements of the collected hormones, the area under the curve with respect to increase will be calculated and used as a dependent variable [71]. As an additional effort to ensure that participants with a delayed significant increase in their hormones will not be excluded, an adjusted increase value will be calculated, regardless of the time of measurement. This value will be obtained by subtracting the baseline value from the peak value. Multiple regressions will be calculated again with these adjusted values. Nonbiological dependent variables, such as those obtained from the psychological test battery, will be added as means. Only data from individuals who have fully completed the TSST protocol will be included in the final analyses. Missing data will be added by multiple imputation.

### Explorative Statistical Analysis

There are exploratory analyses planned on the association of self-reported GRSCs with social motives and their moderating role in psychobiological responses to social support. No further subgroup analyses are planned.

### Monitoring

#### Data Monitoring

In addition to the 2 principal investigators, who are in constant communication about data, no other data monitoring committee is required.

#### Description of Interim Analysis and Stopping Guidelines

No interim analysis or guidelines for study termination are provided. Data collection will cease when the target sample size is reached.

#### Harms

No adverse side effects have been reported with the TSST protocol. The experimenters will collect spontaneously reported adverse events and ask participants at the end of the experiment explicitly whether adverse events or unintended effects occurred.

## Results

The Ethics Committee of the University of Constance approved the application to conduct the study on December 18, 2018. The start of the experiment was planned for the beginning of 2019 but was postponed to June 2021 due to COVID-19. The protocol version is dated May 22, 2022. Data collection will take place until the end of 2022. Publication of the initial results is planned for spring 2023.

## Discussion

The aim of this study is to investigate whether social motives and participants’ sex moderate the effect of social support in stressful situations. We expect that participants with a strong affiliation motive will benefit from social support in terms of reduced psychobiological stress responses when being critically evaluated by others. For those participants, social support is supposed to serve as a stress buffer. In contrast, social support is expected to act as a stress amplifier in participants with a high-power motive, resulting in higher psychobiological stress responses.

To elucidate the influence of implicit social motives on affect in specific situations, we will record participants’ motive-specific affect. We postulate that participants with a high affiliation motive, will respond to social support with affiliation and an increase in progesterone. Participants with a high power motive will show feelings of inferiority and a decrease in testosterone or estradiol.

To explore the influence of implicit motives without bias, we also consider sex as a variable in our study. It is hypothesized that males exhibit a higher power motive and females exhibit a higher affiliation motive. This is expected to be reflected in motive-specific hormones.

Some studies have shown that social support has positive effects [72], while others have reported no, small, or adverse effects of social support [73,74]. With the introduction of implicit motives as moderator variables, as well as taking sex into account, we strive to explain why people react differently in same situations. Through this person-situation approach, we enable a more differentiated view on the effect of social support in stress situations. In summary, with this sophisticated view, we aim to provide a foundation that interventions could be designed in an individualized way and therefore only produce positive effects and no adverse effects.

A broad investigation with induced stress, standardized social support, and the assessment of implicit motives has not been performed in any study known to us. Furthermore, we will cover a large spectrum of methods with our planned study. In addition to self-reports (psychological test battery) and ratings by third parties (ratings of speech performance), we will additionally assess a variety of physiological parameters (hormones, heart rate, and heart rate variability). Therefore, this study lays a comprehensive foundation for further gainful research.

The lack of a control group receiving no stress induction (placebo TSST) could be considered a limitation of the study. However, since the TSST is an established procedure that reliably elicits stress, we believe that a control group can be avoided for pragmatic reasons [51]. In addition, the participants, as well as the research team, will wear FFP2 masks throughout the experiment owing to COVID-19. The effect of mask wearing on the TSST is difficult to assess and will need to be observed.

## Appendix

Multimedia Appendix 1 Screening online survey.

Multimedia Appendix 2 Study information sheet for the laboratory session.

Multimedia Appendix 3 Study protocol.

Multimedia Appendix 4 Daily screening questionnaire.

Multimedia Appendix 5 Instructions for participants.

Multimedia Appendix 6 Social support instructions for the associate.

Multimedia Appendix 7 Procedure for the panel.

Multimedia Appendix 8 Manipulation check for social support.

Multimedia Appendix 9 Debriefing form.

Multimedia Appendix 10 Test battery of psychological questionnaires.

Multimedia Appendix 11 Instructions for the collection and processing of saliva samples.

Multimedia Appendix 12 Peer-review report by the Deutsche Forschungsgemeinschaft (German Research Foundation).

## Abbreviations

GRSC: Gender Role Self-Concept
MDBF: Multidimensional Well-Being Questionnaire
PSE: Picture Story Exercise
TSST: Trier Social Stress Test
TSST-G: Trier Social Stress Test for Groups

## Data Availability

The study protocol has been preregistered (Open Science Framework preregistration: registration DOI 10.17605/OSF.IO/984RW) [75]. Data and statistical codes will be published on Open Science Framework as they become available.

The data will still be accessible after publication by choosing publishing houses that support an open data policy. Furthermore, we will inform researchers about the openness of our data (eg, state repositories in publications and aim to publish in journals that use badges for open data) and explicitly invite members of our specific scientific disciplines to reuse the data (ie, in collaborations, at conferences, in talks, and for poster presentations).

## References

[1] McEwen BS (2000). The neurobiology of stress: from serendipity to clinical relevance. Brain Research.

[2] Sapolsky R, Romero L, Munck A (2000). How do glucocorticoids influence stress responses? Integrating permissive, suppressive, stimulatory, and preparative actions. Endocr Rev.

[3] Pemberton R, Fuller Tyszkiewicz MD (2016). Factors contributing to depressive mood states in everyday life: A systematic review. J Affect Disord.

[4] Feldman PJ, Cohen S, Lepore SJ, Matthews KA, Kamarck TW, Marsland AL (1999). Negative emotions and acute physiological responses to stress. Ann Behav Med.

[5] World Health Organization.

[6] Hobfoll SE, Stokes JP, Duck S, Hay DF, Hobfoll SE, Ickes W, Montgomery BM (1988). The process and mechanics of social support. Handbook of personal relationships: Theory, research and interventions.

[7] Berkman LF, Glass T, Brissette I, Seeman TE (2000). From social integration to health: Durkheim in the new millennium. Social Science & Medicine.

[8] Ditzen B, Heinrichs M (2014). Psychobiology of social support: the social dimension of stress buffering. Restor Neurol Neurosci.

[9] Pressman SD, Cohen S (2005). Does positive affect influence health?. Psychol Bull.

[10] Uchino B (2004). Social support and physical health: Understanding the health consequences of relationships.

[11] Cohen S, Wills TA (1985). Stress, social support, and the buffering hypothesis. Psychological Bulletin.

[12] Broadhead W, Kaplan B, James S, Wagner E, Schoenbach V, Grimson R, Heyden S, Tibblin G, Gehlbach SH (1983). The epidemiologic evidence for a relationship between social support and health. Am J Epidemiol.

[13] Brummett BH, Barefoot JC, Siegler IC, Clapp-Channing NE, Lytle BL, Bosworth HB, Williams RB, Mark DB (2001). Characteristics of socially isolated patients with coronary artery disease who are at elevated risk for mortality. Psychosom Med.

[14] Rutledge T, Reis SE, Olson M, Owens J, Kelsey SF, Pepine CJ, Mankad S, Rogers WJ, Bairey Merz CN, Sopko G, Cornell CE, Sharaf B, Matthews KA, National Heart‚ Lung‚Blood Institute (2004). Social networks are associated with lower mortality rates among women with suspected coronary disease: the National Heart, Lung, and Blood Institute-Sponsored Women's Ischemia Syndrome Evaluation study. Psychosom Med.

[15] Kulik JA, Mahler HI (1989). Social support and recovery from surgery. Health Psychology.

[16] Bianco T, Eklund R (2001). Conceptual considerations for social support research in sport and exercise settings: The case of sport injury. Journal of Sport and Exercise Psychology.

[17] Schultheiss OC, John OP, Robins RW, Pervin LA (2008). Implicit motives. Handbook of Personality: Theory and Research, 3rd Edn.

[18] Brunstein JC, Schultheiss O, Brunstein J (2010). Implicit motives and explicit goals: The role of motivational congruence in emotional well-being. Implicit Motives.

[19] McClelland D (1985). Human motivation.

[20] McClelland DC, Koestner R, Weinberger J (1989). How do self-attributed and implicit motives differ?. Psychological Review.

[21] Schüler J, Brandstätter V, Wegner M, Baumann N (2015). Testing the convergent and discriminant validity of three implicit motive measures: PSE, OMT, and MMG. Motiv Emot.

[22] Schultheiss O, Brunstein J (2010). Implicit Motives.

[23] Dufner M, Arslan RC, Hagemeyer B, Schönbrodt FD, Denissen JJA (2015). Affective contingencies in the affiliative domain: Physiological assessment, associations with the affiliation motive, and prediction of behavior. J Pers Soc Psychol.

[24] French EG (1956). Motivation as a variable in work-partner selection. J Abnorm Psychol.

[25] Atkinson JW, Heyns RW, Veroff J (1954). The effect of experimental arousal of the affiliation motive on thematic apperception. The Journal of Abnormal and Social Psychology.

[26] Weinberger J, Cotler T, Fishman D (2010). The Duality of Affiliative Motivation. Implicit Motives.

[27] Hofer J, Hagemeyer B, Heckhausen J, Heckhausen H (2018). Soziale Anschlussmotivation: Affiliation und Intimität. Motivation und Handeln.

[28] Winter DG (1973). The power motive.

[29] Schmalt H, Heckhausen H, Heckhausen J, Heckhausen H (2006). Machtmotivation. Motivation und Handeln.

[30] Fodor E, Schultheiss O, Brunstein J (2010). Power Motivation. Implicit Motives.

[31] Baumann N, Kaschel R, Kuhl J (2005). Striving for unwanted goals: stress-dependent discrepancies between explicit and implicit achievement motives reduce subjective well-being and increase psychosomatic symptoms. J Pers Soc Psychol.

[32] Hofer J, Busch H (2011). When the needs for affiliation and intimacy are frustrated: Envy and indirect aggression among German and Cameroonian adults. Journal of Research in Personality.

[33] Schultheiss O (2013). The hormonal correlates of implicit motives. Social and Personality Psychology Compass.

[34] Schultheiss OC, Köllner MG, John OP, Robins RW (2021). Implicit motives. Handbook of personality: Theory and research.

[35] Stanton SJ, Schultheiss OC (2009). The hormonal correlates of implicit power motivation. J Res Pers.

[36] Schultheiss OC, Wirth MM, Stanton SJ (2004). Effects of affiliation and power motivation arousal on salivary progesterone and testosterone. Horm Behav.

[37] Wirth MM, Schultheiss OC (2006). Effects of affiliation arousal (hope of closeness) and affiliation stress (fear of rejection) on progesterone and cortisol. Horm Behav.

[38] Schüler J, Job V, Fröhlich SM, Brandstätter V (2009). Dealing with a ‘hidden stressor’: emotional disclosure as a coping strategy to overcome the negative effects of motive incongruence on health. Stress and Health.

[39] Brandstätter V, Job V, Schulze B (2016). Motivational incongruence and well-being at the workplace: person-job fit, job burnout, and physical symptoms. Front Psychol.

[40] Ditzen B, Neumann ID, Bodenmann G, von Dawans B, Turner RA, Ehlert U, Heinrichs M (2007). Effects of different kinds of couple interaction on cortisol and heart rate responses to stress in women. Psychoneuroendocrinology.

[41] Kirschbaum C, Klauer T, Filipp S, Hellhammer DH (1995). Sex-specific effects of social support on cortisol and subjective responses to acute psychological stress. Psychosom Med.

[42] Antonucci TC, Akiyama H (1987). An examination of sex differences in social support among older men and women. Sex Roles.

[43] Jackson T (2006). Relationships between perceived close social support and health practices within community samples of American women and men. The Journal of Psychology.

[44] Knoll N, Schwarzer R Gender and Age Differences in Social Support: A Study of East German Migrants. Freie Universität Berlin.

[45] Denzinger F, Backes S, Job V, Brandstätter V (2016). Age and gender differences in implicit motives. Journal of Research in Personality.

[46] Drescher A, Schultheiss OC (2016). Meta-analytic evidence for higher implicit affiliation and intimacy motivation scores in women, compared to men. Journal of Research in Personality.

[47] Pang JS, Schultheiss OC (2005). Assessing implicit motives in U.S. college students: effects of picture type and position, gender and ethnicity, and cross-cultural comparisons. J Pers Assess.

[48] Athenstaedt U (2016). On the content and structure of the gender role self-concept: including gender-stereotypical behaviors in addition to traits. Psychology of Women Quarterly.

[49] von Dawans B, Kirschbaum C, Heinrichs M (2011). The Trier Social Stress Test for Groups (TSST-G): A new research tool for controlled simultaneous social stress exposure in a group format. Psychoneuroendocrinology.

[50] Kirschbaum C, Pirke K, Hellhammer DH (2008). The ‘Trier Social Stress Test’ – A tool for investigating psychobiological stress responses in a laboratory setting. Neuropsychobiology.

[51] Dickerson SS, Kemeny ME (2004). Acute stressors and cortisol responses: a theoretical integration and synthesis of laboratory research. Psychol Bull.

[52] Schultheiss OC, Wiemers US, Wolf OT (2014). Implicit need for achievement predicts attenuated cortisol responses to difficult tasks. J Res Pers.

[53] Wiemers US, Schultheiss OC, Wolf OT (2015). Public speaking in front of an unreceptive audience increases implicit power motivation and its endocrine arousal signature. Horm Behav.

[54] Liu JJ, Ein N, Peck K, Huang V, Pruessner JC, Vickers K (2017). Sex differences in salivary cortisol reactivity to the Trier Social Stress Test (TSST): A meta-analysis. Psychoneuroendocrinology.

[55] Goodman WK, Janson J, Wolf JM (2017). Meta-analytical assessment of the effects of protocol variations on cortisol responses to the Trier Social Stress Test. Psychoneuroendocrinology.

[56] Robles TF (2007). Stress, social support, and delayed skin barrier recovery. Psychosom Med.

[57] Miller R, Plessow F, Kirschbaum C, Stalder T (2013). Classification criteria for distinguishing cortisol responders from nonresponders to psychosocial stress: evaluation of salivary cortisol pulse detection in panel designs. Psychosom Med.

[58] Faul F, Erdfelder E, Buchner A, Lang A (2009). Statistical power analyses using G*Power 3.1: tests for correlation and regression analyses. Behav Res Methods.

[59] Foley P, Kirschbaum C (2010). Human hypothalamus-pituitary-adrenal axis responses to acute psychosocial stress in laboratory settings. Neurosci Biobehav Rev.

[60] Schultheiss OC, Pang JS, Robins RW, Fraley RC, Krueger RF (2007). Measuring implicit motives. Handbook of research methods in personality psychology.

[61] Zimmermann F, Sieverding M, Müller SM (2010). Gender-related traits as predictors of alcohol use in male German and Spanish university students. Sex Roles.

[62] Fuchs R, Klaperski S, Gerber M, Seelig H (2015). Messung der Bewegungs- und Sportaktivität mit dem BSA-Fragebogen. Zeitschrift für Gesundheitspsychologie.

[63] Schulz U, Schwarzer R (2003). Soziale Unterstützung bei der Krankheitsbewältigung: Die Berliner Social Support Skalen (BSSS). Diagnostica.

[64] Steyer R (1997). Der Mehrdimensionale Befindlichkeitsfragebogen MDBF [Multidimensional mood questionnaire].

[65] Spielberger CD (1970). Manual for the State-trait Anxiety Inventory.

[66] Job V, Bernecker K, Dweck CS (2012). Are implicit motives the need to feel certain affect? Motive-affect congruence predicts relationship satisfaction. Pers Soc Psychol Bull.

[67] Ryan RM, Deci EL (2000). Self-determination theory and the facilitation of intrinsic motivation, social development, and well-being. American Psychologist.

[68] Bernecker K, Job V (2010). Assessing implicit motives with an online version of the picture story exercise. Motiv Emot.

[69] Winter D (1994). Manual for Scoring Motive Imagery in Running Text (Version 4.2).

[70] Ascheron C (2019). Wissenschaftliches Publizieren und Präsentieren Ein Praxisleitfaden mit Hinweisen zur Promotion und Karriereplanung.

[71] Pruessner JC, Kirschbaum C, Meinlschmid G, Hellhammer DH (2003). Two formulas for computation of the area under the curve represent measures of total hormone concentration versus time-dependent change. Psychoneuroendocrinology.

[72] Abraído-Lanza AF (2004). Social support and psychological adjustment among Latinas with arthritis: a test of a theoretical model. Ann Behav Med.

[73] Bolger N, Zuckerman A, Kessler RC (2000). Invisible support and adjustment to stress. Journal of Personality and Social Psychology.

[74] Collins NL, Dunkel-Schetter C, Lobel M, Scrimshaw SC (1993). Social support in pregnancy: Psychosocial correlates of birth outcomes and postpartum depression. Journal of Personality and Social Psychology.

[75] Schüler J, Ditzen B, Haufler A Social support as a stress buffer or stress amplifier: The moderating role of social motives. Open Science Framework.

