# Metadata Correction: Social Support as a Stress Buffer or Stress Amplifier and the Moderating Role of Implicit Motives: Protocol for a Randomized Study

**DOI:** 10.2196/42713

**Published:** 2022-10-25

**Authors:** Alisa Haufler, Beate Ditzen, Julia Schüler

**Affiliations:** 1 Department of Sport Science University of Konstanz Constance Germany; 2 Institute for Medical Psychology Heidelberg University Hospital Heidelberg Germany; 3 Heidelberg University Heidelberg Germany

In “Social Support as a Stress Buffer or Stress Amplifier and the Moderating Role of Implicit Motives: Protocol for a Randomized Study” ([JMIR Res Protoc 2022;11(8):e39509]) the authors noted three errors in the metadata.

First, in the originally published article, the authorship order was incorrect. The article was originally published with the following authorship order:

Julia Schüler, Alisa Haufler, Beate Ditzen

In the corrected article, the authorship order has been updated as follows:

Alisa Haufler, Beate Ditzen, Julia Schüler

Second, *Beate Ditzen* was listed as the Corresponding Author. However, the Corresponding Author has now been changed to *Julia Schüler*.

Third, one affiliation was incomplete and one affiliation was omitted for author Beate Ditzen. This author's affiliation was originally published as follows:

^2^Institute for Medical Psychology, Heidelberg, Germany

Beate Ditzen's affiliations have now been updated to:

^2^Institute for Medical Psychology, Heidelberg University Hospital, Heidelberg, Germany

^3^Heidelberg University, Heidelberg, Germany

The correction will appear in the online version of the paper on the JMIR Publications website on October 25, 2022, together with the publication of this correction notice. Because this was made after submission to PubMed, PubMed Central, and other full-text repositories, the corrected article has also been resubmitted to those repositories.

