# Supplementary material for: Social Support as a Stress Buffer or Stress Amplifier and the Moderating Role of Implicit Motives: Protocol for a Randomized Study
Source: JMIR Res Protoc. 2022 Aug 9;11(8):e39509. doi: 10.2196/39509 (PMC9399871; doi:10.2196/39509)
Supplement: Multimedia Appendix 1 [file resprot_v11i8e39509_app1.docx]

Screening online survey

Study Information Sheet + Informed Consent

Verbal creativity and physiological responses

Welcome and thank you for participating in our study on "verbal creativity and physiological responses." We are trying to find out how creativity in writing stories is related to creativity in describing oneself and how these two factors influence physiological parameters.

In the first part of the study (the current online survey), we will ask you to write short creative stories to six pictures and to complete some questions about yourself. This part will take about 30 minutes.

At the end of the questionnaire, you will be asked to provide your e-mail address. If you are suitable for our study, we will propose you a date for the second part of the study via this e-mail.

The second part of this study will take place in the laboratory. Here, we will measure your heartbeat over the duration of the study and take saliva samples at various times. We will ask you to perform a fictional job interview in front of a panel consisting of two people, followed by a small cognitive task. During this, you will be filmed as well as audio recorded. This part will be about 90 minutes. Afterwards you will receive a payment of 30 Euro in cash.

Please read the following points carefully:

- All data collected in this study will be kept strictly confidential. Your data will be anonymized so that no conclusion can be drawn about your person.
- Your personal data will only be passed on in anonymized form to outside institutions for research purposes.
- The responsible experts of the authorities and the ethics committee may inspect the original data for testing and control purposes, but under strict observance of confidentiality.
- You can withdraw you´re consent to participate at any time and without giving any reason without any disadvantage.
- We would like to draw your attention to the fact that stress reactions of a physical nature and in sensation may occur during the conduct of the study. Therefore, please only participate in the study if you have no previous illnesses and feel healthy and vital.

If you have any questions, please feel free to contact Studie_Verbale_Kreativität_und_Leistung@uni-konstanz.de or the investigator in the laboratory part of the study. Please click "continue" **if you understand everything**.

--------------------------------------------------------next page in Limesurvey ----------------------------------------------

By pressing the **"Next"** button, you confirm that you have been informed about the following points. Please take your time for this and read through everything.

Declaration of consent:

- I know that my personal data will only be passed on in anonymized form to outside institutions for research purposes.
- I have been informed in written form by the study management about the aims, the procedure of the study, the expected effects, possible advantages and disadvantages, as well as possible risks.
- I have read and understood the information about the study.
- I have had the opportunity to ask questions in written form.
- I agree that the responsible experts of the authorities and the ethics committee may inspect my original data for testing and control purposes, but under strict observance of confidentiality.
- I am participating in this study voluntarily.
- I may withdraw my consent to participate at any time and without giving any reason without any disadvantage to me.
- I have had sufficient time to make my decision. By clicking on the "Continue" button, I declare that I have been sufficiently informed about the study and give my consent to participate in the study.

PSE Pictures

| **Couple by the river**  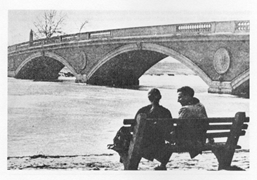 |  | **Beachcombers** 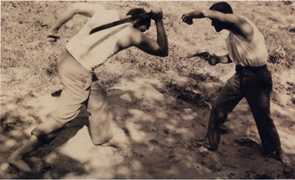 |
| --- | --- | --- |
| **Nightclub scene**  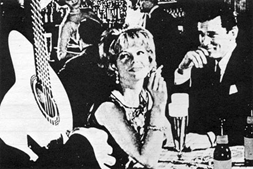 |  | **Newpic32**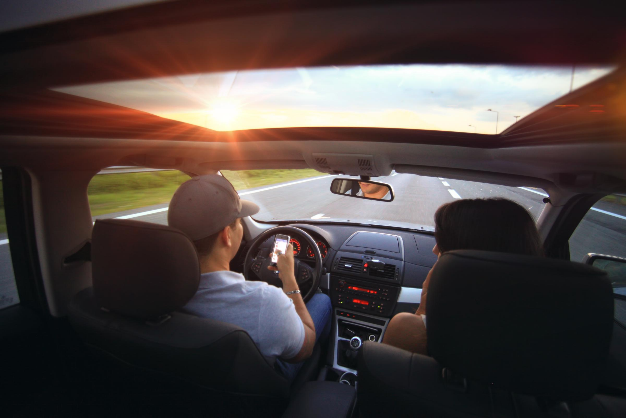 |
| **Sorrow**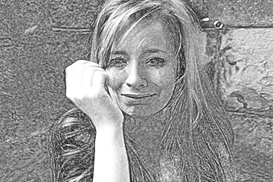 |  | **Newpic9**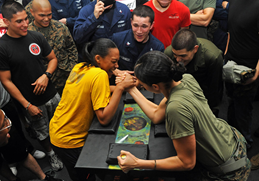 |

*Note****.*** Schönbrodt, F. D., Hagemeyer, B., Brandstätter, V., Czikmantori, T., Gröpel, P., Hennecke, M., . . . Schultheiss, O. C. (2020). Measuring implicit motives with the picture story exercise (pse): Databases of expert-coded german stories, pictures, and updated picture norms. *Journal of Personality Assessment, Advance online publication*. doi:10.1080/00223891.2020.1726936. Pictures available at: https://osf.io/pqckn/wiki/home/

Screening Questionnaire

**Thank you for agreeing to participate in our study.**

In the following, we will ask you to answer some questions about yourself. Your data will of course be kept **strictly confidential** and **anonymous**!

Please generate your personal participant code here. It is very important that you generate it according to the following rules so that we can process your data anonymously.

To generate your code please enter: Example

the first letter of your mother's first name, A (Astrid)

the last letter of your father's first name, T (Helmut)

your month of birth as a number, 04 (April)

and the first letter of your first name. S (Simon)

Generated example code: AT04S

**1. How old are you?**

_ _ years

**2. Gender:**

women  men  diverse

**3**. **How tall are you (please provide whole numbers, no ranges of numbers)?**

_ _ _ cm

**4. How much do you weigh (please provide whole numbers, no ranges of numbers)?**

_ _ _ kilograms

**5. Do you have a job (including voluntary and part-time jobs)?**

yes  no

**If no, please continue with question 6**

**5.1. If yes: Please state the job title.**

**6. Are you studying?**

yes  no

**If no, please continue with question 7.**

**6.1. If yes: Please name your major field of study.**

**6.2. If yes: What semester are you in?**

**7. Do you suffer from one or more (chronic) physical diseases (e.g. cardiovascular complaints, diabetes, neurological diseases, skin diseases, circulatory disorders, immune system disorders, endocrine diseases, etc.)?**

yes  no

**If no, please continue with question 8.**

**7.1. If yes: Which chronic disease(s)?**

**8. Do you suffer from a medically or psychotherapeutically diagnosed mental disorder (e.g. depression, sleep disorders) and /or a diagnosed psychiatric or neurological disorder (e.g. disorders of attention)?**

yes  no

**If no, please continue with question 9.**

**8.1. Which disorder(s) and/or disease(s)?**

**9. Do you suffer from allergies?**

yes  no

**If no, please continue with question 10.**

**9.1. If yes: Which allergies do you suffer from? Please name them briefly.**

**10. In the last three months, have you been treated in a hospital (operations, acute care) or had an operation in the oral cavity by a dentist / surgeon?**

yes  no

**If no, please continue with question 11.**

**10.1. If yes: How many months and days ago did this/these procedure(s) take place and what was it/they were (please indicate whole numbers, no number ranges)?**

Intervention____________________ took place _ _ months and_ _ days ago.

Intervention____________________ took place _ _ months and_ _ days ago.

Intervention____________________ took place _ _ months and_ _ days ago.

Intervention____________________ took place _ _ months and_ _ days ago.

**11. In the last year, have you experienced one or more massive changes in your life circumstances (e.g., moving, new job, marriage, divorce, birth of a child, death of a close friend or relative, etc.)?**

yes  no

**If no, please continue with question 12.**

**11.1. If yes: What were the changes? Please describe them briefly.**

**12. Do you take - besides hormonal contraceptives - another hormone preparation (e.g. thyroid hormones, general hormone substitution etc.)?**

yes  no

**If no, please continue with question 13.**

**12.1. If yes: Please explain in more detail (product name):**

**13. Do you take psychotropic drugs?**

yes  no

**If no, please continue with 14.**

**13.1. If yes: Which psychotropic drug(s) are you taking (product name(s)):**

**14. Do you regularly take medications that are not psychotropic or hormonal drugs?**

yes  no

**If no, please continue with 15.**

**14.1. If yes: What is the medication(s) (product name(s))?**

**15. Have you smoked regularly in your life for a period of more than 6 months? By regular, we mean at least 1 pack of cigarettes per month.**

yes  no

**If no, please continue with 16.**

**15.1. If yes: Do you currently smoke, even if only occasionally?**

yes  no

**16. Do you consume caffeine?**

yes  no

**17. How often do you drink alcohol?**

never

about 1 time per month

2 - 4 times a month

2 - 3 times a week

4 times a week or more

**18. Do you regularly use narcotics ("drugs")?**

yes  no

**If no, please continue with 19.**

**18.1. If yes: Which narcotic(s) are you using?**

**19.** **Do you regularly perform meditation, mindfulness or relaxation exercises?**

yes  no

**20. Have you ever participated in a study in which you faced a panel of two people in white lab coats?**

yes  no

**21. Your employment or your training includes...**

seated activities  none  rather little  rather more  much

moderate movements  none  rather little  rather more  much

intensive movements  none  rather little  rather more  much

**22. On how many days and how long did you engage in the following activities during the last 4 weeks?**

| Walking to the workplace (also part of the route) | on ...... days during the last 4 weeks | ca. ...... minutes per day | didn’t do that |
| --- | --- | --- | --- |
| Walking for shopping | on ...... days during the last 4 weeks | ca. ...... minutes per day | didn’t do that |
| Biking to the workplace | on ...... days during the last 4 weeks | ca. ...... minutes per day | didn’t do that |
| Biking for other transportation reasons | on ...... days during the last 4 weeks | ca. ...... minutes per day | didn’t do that |
| Going for a walk | on ...... days during the last 4 weeks | ca. ...... minutes per day | didn’t do that |
| Gardening (e.g., mowing the lawn, trimming hedges) | on ...... days during the last 4 weeks | ca. ...... minutes per day | didn’t do that |
| Strenuous homework (e.g., cleaning, tidying) | on ...... days during the last 4 weeks | ca. ...... minutes per day | didn’t do that |
| Strenuous care (e.g., care for the elderly) | on ...... days during the last 4 weeks | ca. ...... minutes per day | didn’t do that |

**23. On how many days and how long did you engage in the following activities during the last 4 weeks?**

Climbing stairs

on ...... days during the last 4 weeks ca. ...... floors per day  didn’t do that

**24. Did you engage in regular exercise or sport activities during the last 4 weeks?**

yes - continue with question 24  no - continue with question 25

**25. What kind of exercise or sport activities did you perform?**

Please consider cycling here only if it is performed as a sporting activity (cycling for work and other transport purposes please enter above under question 3).

| **Activity A**  .........................................  (please note here) | **Activity B**  .........................................  (please note here) | **Activity C**  .........................................  (please note here) |
| --- | --- | --- |
| I performed activity **A** during the **last 4 weeks**  about ..... times, and each time for about ...... minutes | I performed activity **B** during the **last 4 weeks**  about ..... times, and each time for about ...... minutes | I performed activity **C** during the last **4 weeks**  about ..... times, and each time for about ...... minutes |

*Note.* 21-25: Fuchs, R., Klaperski, S., Gerber, M., & Seelig, H. (2015). Messung der Bewegungs-und Sportaktivität mit dem BSA-Fragebogen [Measurement of physical activity and sports activity with the BSA questionnaire]. *Zeitschrift für Gesundheitspsychologie*.

**For female participants::**

**26. Do you have a menstrual period?**

yes  no

**If no, please continue with 27.**

**If yes, please continue with question 26.1.**

**26.1. When was the start of your last menstrual period (or period) (please give whole numbers, no number ranges)?**

_ _ days ago.

**26.2. Do you have your menstrual bleeding (or period) at regular intervals (are the time intervals between the start of your menstrual bleeding the same)?**

yes  no

**26.3. How long does your menstrual cycle usually last?**

less then 23 days

23-25 days

26-28 days

29-31 days

32-34 days

35-37 days

longer than 37 days

**26.4. How long does your menstruation (or period) usually last?**

1–2 days

3–4 days

5–6 days

7–8 days

more than 8 days

**27. Do you take a "birth control pill" for hormonal contraception?**

yes  no

**If no, please continue with 23.**

**27.1. If yes: Please explain in more detail (product name):**

**28. Do you use a hormonal contraceptive other than the "birth control pill" (e.g., vaginal ring, contraceptive patches and sticks, hormonal IUD, depot preparations (such as the three-month injection)?**

no  yes

**29. Is there currently a pregnancy?**

yes  no

**30. If you are already a mother, are you currently breastfeeding?**

yes  no

**31. How do you see yourself? Some characteristics are listed below. Please indicate on the scale from 1 ("never" or "almost never") to 7 ("always" or "almost always") to what extent each characteristic applies to you.**

|  | 1 | 2 | 3 | 4 | 5 | 6 | 7 |  |  |  |  |  |  |  |
| --- | --- | --- | --- | --- | --- | --- | --- | --- | --- | --- | --- | --- | --- | --- |
| defend my opinion |  |  |  |  |  |  |  |  |  |  |  |  |  |  |
| affectionate |  |  |  |  |  |  |  |  |  |  |  |  |  |  |
| independent |  |  |  |  |  |  |  |  |  |  |  |  |  |  |
| sensitive to the needs of others |  |  |  |  |  |  |  |  |  |  |  |  |  |  |
| assertive |  |  |  |  |  |  |  |  |  |  |  |  |  |  |
| tender |  |  |  |  |  |  |  |  |  |  |  |  |  |  |
| strong personality |  |  |  |  |  |  |  |  |  |  |  |  |  |  |
| warm-hearted |  |  |  |  |  |  |  |  |  |  |  |  |  |  |
| forceful |  |  |  |  |  |  |  |  |  |  |  |  |  |  |
| Eager to soothe hurt feelings |  |  |  |  |  |  |  |  |  |  |  |  |  |  |
| have leadership abilities |  |  |  |  |  |  |  |  |  |  |  |  |  |  |
| gentle |  |  |  |  |  |  |  |  |  |  |  |  |  |  |
| willingness to take risks |  |  |  |  |  |  |  |  |  |  |  |  |  |  |
| understanding |  |  |  |  |  |  |  |  |  |  |  |  |  |  |
| dominant |  |  |  |  |  |  |  |  |  |  |  |  |  |  |
| compassionate |  |  |  |  |  |  |  |  |  |  |  |  |  |  |

*Note.* 31: Zimmermann, F., Sieverding, M., & Müller, S. M. (2011). Gender-related traits as predictors of alcohol use in male German and Spanish university students. *Sex Roles, 64*, 394-404. doi:10.1007/s11199-010-9897-9
