# Supplementary material for: Social Support as a Stress Buffer or Stress Amplifier and the Moderating Role of Implicit Motives: Protocol for a Randomized Study
Source: JMIR Res Protoc. 2022 Aug 9;11(8):e39509. doi: 10.2196/39509 (PMC9399871; doi:10.2196/39509)
Supplement: Multimedia Appendix 2 [file resprot_v11i8e39509_app2.docx]

**Study information sheet for the laboratory session**

Study information sheet

Verbal creativity and physiological responses

Welcome and thank you for participating in our study on "verbal creativity and physiological responses." We are trying to find out how creativity in writing stories is related to creativity in describing oneself and how these two factors influence physiological parameters.

The first part of the study has already been completed. Now the second part of the study is taking place in the laboratory. This part will be about 90 minutes. Afterwards you will receive a payment of 30 Euro in cash.

We will measure your heartbeat over the duration of the study and take saliva samples at various times. We will ask you to perform a fictional job interview in front of a panel consisting of two people, followed by a small cognitive task. During this, you will be filmed as well as audio recorded.

Please read the following points carefully:

- All data collected in this study will be kept strictly confidential. Your data will be anonymized so that no conclusion can be drawn about your person.
- Your personal data will only be passed on in anonymized form to outside institutions for research purposes.
- The responsible experts of the authorities and the ethics committee may inspect the original data for testing and control purposes, but under strict observance of confidentiality.
- You can withdraw you´re consent to participate at any time and without giving any reason without any disadvantage.
- We would like to draw your attention to the fact that stress reactions of a physical nature and in sensation may occur during the conduct of the study. Therefore, please only participate in the study if you have no previous illnesses and feel healthy and vital.

If you have any questions, please feel free to contact the investigator in the laboratory.

Informed consent

Informed consent to take part in a study of the Division of Sports Science, Sports Psychology.

- Please read this form carefully.
- Please ask the investigator if there is anything you do not understand or would like to know.

**Title of the study: "Verbal creativity and physiological responses"**

**Head of study:** Prof. Dr. Julia Schüler, University of Konstanz

Department of Sports Science, Sport Psychology, Universitätsstraße 10, 78464 Konstanz, Germany; e-mail. julia.schueler@uni-konstanz.de; phone. + 49 7531 88 2629

**Location of the study:** Sport Psychology Laboratory of the University of Konstanz in the Unisporthalle Konstanz (Unisporthalle Konstanz, Mainaustraße 213, 78464 Konstanz)

**Examiner** (= study director; surname and first name):

_______________________________________________________________

**Participant (**name and first name):

_______________________________________________________________

Date of birth: _____________________________________________________________

Please mark with a cross: Man Woman

Please mark with a cross where applicable::

- I have been informed in writing about the aims, the procedure of the study, about the expected effects, about possible advantages and disadvantages as well as about possible risks.
- I have read and understood the written subject information (e.g. study information sheet) provided for the above study. I have had the opportunity to ask questions by e-mail.
- If available: My questions related to participation in this study have been satisfactorily answered.
- I can download the written subject information sheet and will receive a copy of my informed consent form by email if I wish.
- I have had sufficient time to make my decision.
- I know that my personal data will only be passed on in anonymized form to outside institutions for research purposes. I agree that the responsible experts of the authorities and the ethics committee may inspect my original data for testing and control purposes, but under strict observance of confidentiality.
- I am participating in this study voluntarily. I may withdraw my consent to participate at any time and without giving reasons, without any disadvantage to me.
- I am aware that during the study, the requirements and restrictions stated in the study information sheet and the study participation email must be adhered to.
- In the interest of my health, the investigator may exclude me from the study at any time. In addition, I inform the investigator of any medications I am taking (prescribed or purchased independently).
- I hereby confirm the accuracy of my information and consent to study participation.

**Investigator's Acknowledgement:** I hereby affirm that I will fulfill all obligations related to this study. If at any time during the conduct of the study I become aware of any issues that may affect the study participant's willingness to participate in the study, I will inform him/her immediately.

______________________________________

**Signature oft the Investigator**
