# Supplementary material for: Social Support as a Stress Buffer or Stress Amplifier and the Moderating Role of Implicit Motives: Protocol for a Randomized Study
Source: JMIR Res Protoc. 2022 Aug 9;11(8):e39509. doi: 10.2196/39509 (PMC9399871; doi:10.2196/39509)
Supplement: Multimedia Appendix 3 [file resprot_v11i8e39509_app3.docx]

*Study Protocol*

Inclusion criteria:

- Age: participation from 18 years
- Gender: female & male
- Language: mother language German

Exclusion criteria:

- Participation in previous stress experiments (TSST).
- Psychology & sports students from the 5^th^ semester
- Women who use hormonal contraception
- Women who are pregnant or breastfeeding
- Any (chronic) physical diseases: e.g. cardiovascular disorders, diabetes, neurological diseases, skin diseases, circulatory disorders, immune system disorders, endocrine disorders
- Psychopathology: medically or psychotherapeutically diagnosed mental disorder (e.g. depression, sleep disorders) and/or suffering from a diagnosed psychiatric or neurological disease (e.g. disorders of attention)
- Regular use of medication: e.g. psychotropic drugs, hormonal preparations, other medication
- Drug use
- Smoking
- BMI of 30 or more (obesity)

Criteria for the Lab session:

- 24 hours prior to lab appointment no caffeine, no alcohol, no drug intake, no exercise.
- No meal 2 hours before laboratory appointment
- 1 hour before lab appointment no soft drinks
- No chewing gum
- No lipstick
- Check sleep (pay attention to daily cortisol)

Time to conduct the study:

- 17:15-19:45

| **Time** | **Phase/Task** |
| --- | --- |
| **Before the study - Online Questionnaire** | |
|  | 1. Participant recruitment: posters, SONA, first semester lectures |
|  | 1. Send e-mail with link of online questionnaire   Online questionnaire content: (Appendix B)   - Study information sheet - Informed consent - PSE (Picture Sory Exercise) - Screening-questionnaire (eligibility criteria) - Assessment of control variables |
|  | 1. Participant completed online questionnaire:   🡪Check eligibility criteria |
|  | 1. Collect participants |
|  | 1. E-mail to participants with appointment suggestions |
|  | 1. Participant confirms lab session by E-mail, otherwise offer alternative appointment |
|  | 1. 5 days before Lab session reminder E-mail |
|  | 1. Reminder E-mail 1 day before the lab session   Content:   - Request to refrain from exercise, caffeine, alcohol, drugs, smoking - No chewing gum or lipstick before the lab session |
| **General Preparation Phase - Set - Up** | |
| -60min | 1. unlock rooms, switch on lights, ventilate if necessary  2. check room set-up  3. paper strips taped as privacy screen in door with glass strip room 210?  4. check number of bags with salicaps and straw  5. check battery cameras & microphones, use spare battery if necessary  6. check battery of PC/tablets in preparation rooms, switch on, set questionnaires, have charger ready if necessary  7. have Polar sensors and pulse belt ready  8. stop watches, white coats, pens and instructions for participants available?  9. check battery of experimenter tablet, have spare battery ready if necessary  10. check connection of HR(V) software to tablet  11. check battery of social supporters' computers, have charging cable or spare battery ready if necessary  12. payment for participants available?  13. all doors closed? |
| **General Preparation Phase - Study** | |
|  | 1. Gremiums members (GM 1 & GM 2), social supporters (Cof 1 & Cof 2), and experimenter (E) are in room 210. |
| -22min | 1. E gets tablet (for markers of HR(V)), always carries it with her |

| -20min | 1. E gets participant 1 (Pb1) 2. Pb1: Welcome + information not to drink during the study 3. Guide Pb1 to room 211, have him/her sit down: 4. Gives study information sheet, subject code sheet + informed consent to Pb1 5. E shows how to put on the belt 6. E: “*I will be right back and will knock then."* 7. E leaves the room 8. Pb1 puts the belt on and completes the Daily screening questionnaire (Appendix D) on PC |
| --- | --- |
|  | 1. Cof 1 gets participant 2 (Pb2) 2. Pb2: Welcome + information not to drink during the study 3. Guide Pb2 to room 212, have him/her sit down 4. Gives study information sheet, subject code sheet + informed consent to Pb2 5. E shows how to put on the belt 6. E: “*I will be right back and will knock then."* 7. E leaves the room 8. Pb2 puts the belt on and completes the Daily screening questionnaire (Appendix D) on PC |
|  | 1. E gets participant 3 (Pb3) 2. Pb3: Welcome + information not to drink during the study 3. Guide Pb3 to room 213, have him/her sit down: 4. Gives study information sheet, subject code sheet informed consent to Pb3 5. E shows how to put on the belt 6. E: “*I will be right back and will knock then."* 7. E leaves the room 8. Pb3 puts the belt on and completes the Daily screening questionnaire (Appendix D) on PC |
| -15min | 1. E goes back to room 211; **[Knocking!]** 2. Check function of pulse belt (Polar sensor) & transmission to tablet 3. Set marker for start of HR(V) measurement **(T0:HR(V))** 4. E: *"Please remain seated quietly."* 5. E leaves the room and takes subject code sheet with her |
|  | 1. E goes back to room 212; **[Knocking!]** 2. Check function of pulse belt (Polar sensor) & transmission to tablet 3. Set marker for start of HR(V) measurement **(T0:HR(V))** 4. E: *"Please remain seated quietly."* 5. E leaves the room and takes subject code sheet with her |
|  | 1. E goes back to room 213; **[Knocking!]** 2. Check function of pulse belt (Polar sensor) & transmission to tablet 3. Set marker for start of HR(V) measurement **(T0:HR(V))** 4. E: *"Please remain seated quietly."* 5. E leaves the room and takes subject code sheet with her |
| T_0_ -11min | 1. E goes back to room 211; knocking 2. Salvia sample Pb 1   Instructions (I): *"You will find a straw and a small plastic jar in the little bag I am about to hand you. Use the straw to pour saliva into the jar until it is filled to a little more than halfway. After using it, you can dispose of the straws. Collect the saliva at the front of the mouth area and squeeze it through the straw. Try not to blow through the straw when doing this! When you have collected enough saliva, please close the small containers carefully (until it "clicks") and put the saliva sample back into the appropriate bag.* ***It is enough saliva when the container is a little more than half full!*** *You can check in between how much is already in the container and then continue to dispense saliva until the required amount is reached. Also, please be careful to avoid bubbles in the sample”* |
|  | 1. Psychological questionnaires (T0):   E: *"Please complete the following questionnaires on your PC."* |
| T_0_ -11min | 1. E goes back to room 212; knocking 2. Salvia sample Pb 2   Instruction (I) as already described by Pb1   1. Psychological questionnaires **(T0)**:   E: *"Please complete the following questionnaires on your PC.”* |
| T_0_ -11min | 1. E goes back to room 213; knocking 2. Salvia sample Pb 3   Instruction (I) as already described by Pb1   1. Psychological questionnaires **(T0)**:   E: *"Please complete the following questionnaires on your PC.”* |
|  | 1. Female confederate gets ready in room 210 (if experiment with 2 confederates, the second gets ready in room 290) |
| **TSST- G Preparation Phase** | |
| -10min | 1. E goes back to room 211 2. Sets 2nd marker for HR(V) **(T1:HR(V))** 3. Hands out written instructions (Appendix E). Waits for questions 4. E opens door for Cof 1 |
| -10min | 1. Female confederate (Cof1) enters room 211 2. E: "Hello (name confederate)." [Turning to participant]: "This is (name   confederate). She is a student assistant at our department. She will be  present during the 10-minute preparation period if you need help."   1. Confederate (Cof1) greets participant enthusiastically, "Hello I am (name). I'm keeping an eye on the time. But I'm also here for you if you need help. I   have some work to do on the computer, but if I can help you in any way,  please let me know. Just reach out!"   1. E: "Thank you (name confederate)." [turns to Pb], "Now begin to prepare for your speech." 2. E leaves room 211, goes to room 212 3. Cof1 starts her own stopwatch on the compute. 4. Cof1: "The time is running from now on! I wish you a lot of success. And as I said, just talk to me if you need help." 5. Cof1 sits down at computer (at an angle to Pb), behaves quietly, works on table at computer 6. Cof1 gives only passive support for first few minutes; Cof1 speaks only when Pb addresses her (Appendix F) 7. In the second 5min of the preparation phase, Cof1 gives active support according to standardized sentences; she notes Pb's reaction to each of them 8. Minute 5: Cof1: "Well, how's it going? Is there anything else I can help you with?" 9. Minute 7: Cof1: "I've also participated in a study like this before, so I know exactly how you feel. But in the end, it wasn't so bad!" 10. Minute 9: Cof1: "Don't worry, soon you'll be done, and we really appreciate you participating in our study!"   *Note: Social support response should seem as natural as possible: Cof may deviate from standard response if context requires, Additional response options are listed in the table for other possible situations.  If Pb explicitly does not want support, give only standard support responses (minute 5,7,9) |
| -10min | 1. E goes to room 212 2. E Sets 2nd marker for HR(V) in room 212 **(T1:HR(V))** 3. Hands out written instructions (Appendix E). Waits for questions. 4. Starts own stopwatch |

|  | 1. E goes to room 213 2. E Sets 2nd marker for HR(V) in room 213 **(T1:HR(V))** 3. Hands out written instructions (Appendix E). Waits for questions.   *Note If there is a second confederate planned then the E and Cof follow the same steps as for Pb 1 (4-18) |
| --- | --- |
|  | 1. E leaves room (213), goes to room 210 via 290, enters subject code, room number and salivette number in table, waits until Cof comes out of room 211 |

| T_1_ 0min | 1. **Cof1 of room 211** after 10min: *"The preparation time has expired."* 2. Salvia sample Pb1 **(T1)** 3. Instruction (I) is the same as in T_0_ 4. Psychological questionnaires: Cof1: *"Please complete the following questionnaires on your PC.”* 5. Cof1: “*You will now change rooms. In the other room the panel is waiting for you and there you will do your interview. Your notes will stay here. Please do not talk to the other participants and do not make eye contact. Please stand in the place with your room number 1 and wait for the panel's instructions."* 6. Cof1: *"I wish you much success. You can do it!"* 7. Cof1 indicates Pb to change the room, closes the door if necessary. |
| --- | --- |
| T_1_ 0min | 1. When stopwatch of E shows 10 min, she goes to room 212 via 211 [open door quietly; do not disturb Pb1!] 2. E: *“The preparation time has expired.“* 3. Salvia sample Pb2 **(T1)** 4. Instruction (I) is the same as in T_0_ 5. Psychological questionnaires **(T1)**: 6. E: *"Please complete the following questionnaires on your PC.”* 7. E waits until Pb 2 ist done 8. E announces change to room 210, notes remain in room 212: *"We will change rooms now. The panel is waiting for them in the other room and that is where you will have your interview. Your notes will remain here. Please do not talk to the other participants and do not make eye contact."* 9. E takes PB2 to room 210 (via room 211) to place 2, E takes saliva samples of Pb2 and Pb1 with her 10. E: “*Please remain standing here until the panel gives you instructions“* |
| T_1_ 0min | 1. E goes back to room 213 2. E: “*The preparation time has expired.“* 3. Salvia sample Pb3 **(T1)** 4. Instruction (I) is the same as in T_0_ 5. 11. Psychological questionnaires **(T1):** 6. E: *"Please complete the following questionnaires on your PC.”* 7. E waits until Pb 3 is done 8. E announces change to room 210, notes remain in room 213: *"We will change rooms now. The panel is waiting for them in the other room and that is where you will have your interview. Your notes will remain here. Please do not talk to the other participants and do not make eye contact."* 9. E takes PB3 to room 210 to place 3, E takes saliva samples with her 10. E remains in room 210 not visible to Pb, and acts quietly 11. 16. If necessary, E helps Pb 1 to find place 1. |
|  | 1. Cof1 leaves room 211 trough room 212 and 213 |
|  | *Note: If the experiment takes place with Cof1 and Cof2, then again the same procedure in 213 is performed by Cof 2 |
|  | 1. Panel members wait until all participants are in place, show no reaction to Pb |
|  | 1. E sets marker 3 for HR(V) measurement **(T2HR(V))** |
| **TSST-G Speech (job interview) (3x3min)** | |
|  | 1. Panel member 2 (GM 2): *"We will now begin the interviews. As soon as your number is called, please step forward to the mark. Your speech will be recorded by video camera. The camera will be aligned first and you will then be asked to begin your speech. Once all applicants have finished their speech, another task will follow."* |
|  | 1. GM 2 (active member): *"Number X1 [room number 11,12 or 13] step forward and stand exactly at the mark."* |
|  | 1. Panel member 1 (GM 1, passive member): microphone orientation. Turn on. |
|  | 1. GM 1: Align video camera, check, start. Sits down and signals GM 2 to continue with head nod. |
| 1min | 1. GM 2: *"Please start your presentation with your participant number and today's date. Begin now!"* |
|  | GM 1 starts stopwatch 2 (puts it between himself and GM 2, tells GM 2 when time is at 2.45 min) |
|  | 5a. Interruptions/Comments: (see Appendix G) |
|  | 5b. After 3 minutes: if possible, interrupt while speaking.  GM 2: *"Thank you, that's enough."* |
|  | GM 1: Notes speaking time of stopwatch 2, sets it back to 0 |
| 4min | 1. GM 2: *"Number X2 step forward and stand exactly at the mark."* |
|  | 1. Panel member 1 (GM 1, passive member): microphone orientation. Turn on. |
|  | 1. GM 1: Align video camera, check, start. Sits down and signals GM 2 to continue with head nod. |
| 5min | 1. GM 2: *"Please start your presentation with your participant number and today's date. Begin now!"* |
|  | GM 1 starts stopwatch 2 (puts it between himself and GM 2, tells GM 2 when time is at 2.45 min) |
|  | 9a. Interruptions/Comments: (see Appendix G) |
| 8min | 9b. After 3 minutes: if possible, interrupt while speaking.  GM 2: *"Thank you, that's enough.”* |
|  | GM 1: Notes speaking time of stopwatch 2, sets it back to 0 |
|  | 1. GM 2: *"Number X3 step forward and stand exactly at the mark."* |
|  | 1. Panel member 1 (GM 1, passive member): microphone orientation. Turn on. |
|  | 1. GM 1: Align video camera, check, start. Sits down and signals GM 2 to continue with head nod. |
| 9min | 1. GM 2: *"Please start your presentation with your participant number and today's date. Begin now!"* |
|  | GM 1 starts stopwatch 2 (puts it between himself and GM 2, tells GM 2 when time is at 2.45 min) |
|  | 13a. Interruptions/Comments: (see Appendix G) |
| 12min | 13b. After 3 minutes: if possible, interrupt while speaking.  GM 2: *"Thank you, that's enough.* |
|  | GM 1: Notes speaking time of stopwatch 2, sets it back to 0 |
| T_2_ 12min | 1. GM2: Salvia sample (Pb1, 2 & 3) **(T2)**   E hands out salivettes and takes salvia samples with her  Instruction (I) is the same as in T_0_  GM2: “*Please fill in these questionnaires.“***(T2)**  E: Distributes paper and pencil version of the psychological questionnaire.  GM2: “*When you are done, put the questionnaire on the floor in front of you“*  E takes questionnaires with her and enters the answers in the table |
|  | 1. E sets 4.marker for HR(V) measurement **(T3HR(V))** |
| **TSST-G Arithmetic Task (3x 3x30sec)** | |
| 13min | After the end of the interview of all 3 persons (**time 12min**) & saliva delivery transition to the calculation task.  GM2 says indifferently and neutrally: *"We now come to the second task. This is an arithmetic task."* |
| 13min | 1. Explanation by GM 2: "*We will call you again by your numbers. Then you step up to the marker, and count backwards in steps of 17 from the number you were told. You will always be called at random and can take your turn again at any time."* |
|  | 1. GM2: *"Number X2 [11,12 or 13] step forward."* |
|  | 1. GM2: *"Please count backwards in steps of 17 from the number* ***4878****. Do this as quickly and as error-free as you can.*   *If you make a mistake, we will bring it to your attention and you will then have to start over at* ***4878****. Please start now."* |
|  | GM 1 starts stopwatch 1 after 25sec nod to GM 2  GM 2 controls numbers |
|  | 3a. Interruptions by GM 2 (Appendix G) e.g.: *"Speed up a little"* |
|  | 3b. After 30sec: Best to interrupt Pb while he is speaking:  GM 2: *"Thank you, that's enough."*  Panel takes notes. |
|  | GM 1 sets stopwatch 1 to 0. |
|  | 1. GM 2: *"Number X1 step forward."* |
|  | 1. GM 2: *"Please count backwards in steps of 17 from the number* ***4623****. Do this as quickly and as error-free as you can.*   *If you make a mistake, we will call it to your attention and you will then have to start over at* ***4623****. Please start now."* |
|  | GM 1 starts stopwatch 1 after 25sec nod to GM 2  GM 2 controls numbers |
|  | 5a. Interruptions by GM 2 (Appendix G) e.g.: *"Speed up a little"* |
|  | 5b. After 30sec: Best to interrupt Pb while he is speaking:  GM 2: *"Thank you, that's enough."*  Panel takes notes. |
|  | GM 1 sets stopwatch 1 to 0. |
|  | 1. GM 2: *"Number X3 step forward."* |
|  | 1. GM 2: *"Please count backwards in steps of 17 from the number* ***3977****. Do this as quickly and as error-free as you can.*   *If you make a mistake, we will call it to your attention and you will then have to start over at* ***3977****. Please start now."* |
|  | GM 1 starts stopwatch 1 after 25sec nod to GM 2  GM 2 controls numbers |
|  | 7a. Interruptions by GM 2 (Appendix G) e.g.: *"Speed up a little"* |
|  | 7b. After 30sec: Best to interrupt Pb while he is speaking:  GM 2: *"Thank you, that's enough."*  Panel takes notes. |
|  | GM 1 sets stopwatch 1 to 0. |
|  | 1. GM2: *"Number X1 please step forward and count backwards from 3518 in steps of 17."* |
|  | GM 1 starts stopwatch 1 after 25sec nod to GM 2  GM 2 controls numbers |
|  | 8a. Interruptions by GM 2 (Appendix G) |
|  | 8b. After 30sec: Best to interrupt Pb while he is speaking:  GM 2: *"Thank you, that's enough."*  Panel takes notes. |
|  | GM 1 sets stopwatch 1 to 0. |
|  | 1. GM2: "Number X3 please step forward and count backwards from **4742** in steps of 17." |
|  | GM 1 starts stopwatch 1 after 25sec nod to GM 2  GM 2 controls numbers |
|  | 9a. Interruptions by GM 2 (Appendix G) |
|  | 9b. After 30sec: Best to interrupt Pb while he is speaking:  GM 2: *"Thank you, that's enough."*  Panel takes notes. |
|  | GM 1 sets stopwatch 1 to 0. |
|  | 1. GM2: *"Number X2 please step forward and count backwards from* ***3756*** *in steps of 17."* |
|  | GM 1 starts stopwatch 1 after 25sec nod to GM 2  GM 2 controls numbers |
|  | 10a. Interruptions by GM 2 (Appendix G) |
|  | 10b. After 30sec: Best to interrupt Pb while he is speaking:  GM 2: *"Thank you, that's enough."*  Panel takes notes. |
|  | GM 1 sets stopwatch 1 to 0. |
|  | 1. GM2: *"Number X3 please step forward and count backwards from* ***4504*** *in steps of 17."* |
|  | GM 1 starts stopwatch 1 after 25sec nod to GM 2  GM 2 controls numbers |
|  | 11a. Interruptions by GM 2 (Appendix G) |
|  | 11b. After 30sec: Best to interrupt Pb while he is speaking:  GM 2: *"Thank you, that's enough."*  Panel takes notes. |
|  | GM 1 sets stopwatch 1 to 0. |
|  | 1. GM2: *"Number X2 please step forward and count backwards from* ***3280*** *in steps of 17."* |
|  | GM 1 starts stopwatch 1 after 25sec nod to GM 2  GM 2 controls numbers |
|  | 12a. Interruptions by GM 2 (Appendix G) |
|  | 12b. After 30sec: Best to interrupt Pb while he is speaking:  GM 2: *"Thank you, that's enough."*  Panel takes notes. |
|  | GM 1 sets stopwatch 1 to 0. |
|  | 1. GM2: *"Number X1 please step forward and count backwards from* ***4215*** *in steps of 17."* |
|  | GM 1 starts stopwatch 1 after 25sec nod to GM 2  GM 2 controls numbers |
|  | 13a. Interruptions by GM 2 (Appendix G) |
|  | 13b. After 30sec: Best to interrupt Pb while he is speaking:  GM 2: *"Thank you, that's enough."*  Panel takes notes. |
|  | GM 1 sets stopwatch 1 to 0. |
| T_3_ +20min | 1. GM2: Salvia sample (Pb1, 2 & 3) **T3**   E hands out salivettes and takes salvia samples with her  Instruction (I) is the same as in T_0_ |
|  | 1. E sets 5th marker for HR(V) measurement **(T4HR(V))** |
|  | 1. **GM1: Save audio/video files** |
| T_3_ + 20min | 1. E in room 210: "*I will pick you up one by one. Please remain standing until then.“* 2. E turned to Pb1: "Please follow me." 3. E takes Pb1 to room 211 4. In room 211. E: “*Please work on the questionnaire on the PC (Psychological questionnaire* ***T3****). After that, you can rest. I will come back in 10 min."* |
|  | 1. E starts stopwatch (carries stopwatch at all 3 Pb, serves control of time during rest phase) |
|  | 1. E goes back to room 210 addressed to Pb2: *"Please follow me."* 2. E takes Pb2 to room 212 via room 290 and room 213. 3. In room 212. E: “*Please work on the questionnaire on the PC (Psychological questionnaire* ***T3****). After that, you can rest. I will come back in 10 min."* |
|  | 1. E goes back to room 210. 2. E turned to Pb3: "Please follow me." 3. E takes Pb3 to room 213 via room 290. |
|  | 1. In room 213. E: “*Please work on the questionnaire on the PC (Psychological questionnaire* ***T3****). After that, you can rest. I will come back in 10 min."* |
| **Rest- Phase** | |
|  | 1. E goes to room 210 via room 290, waits until stopwatch shows 10min. |
| T_4_+30min | 1. E goes to room 211 2. E sets 6.marker for HR(V) measurement **(T5HR(V))** 3. Salvia sample (Pb 1) **(T4)** 4. Instruction (I) ist the same as in T_0_ 5. Psychological questionnaires: 6. E: “*Please work on the questionnaires on the PC. After that, you can rest. I will come back in 15 min.“* |
| T_4_ +30min | 1. E goes to room 212 2. E sets 6.marker for HR(V) measurement **(T5HR(V))** 3. Salvia sample (Pb 2) **(T4)** 4. Instruction (I) ist the same as in T_0_ 5. Psychological questionnaires **(T4)**: 6. E: “*Please work on the questionnaires on the PC. After that, you can rest. I will come back in 15 min.“* |
| T_4_ +30min | 1. E goes to room 213. 2. E sets 6.marker for HR(V) measurement **(T5HR(V))** 3. Salvia sample (Pb 3) **(T4)** 4. Instruction (I) ist the same as in T_0_ 5. Psychological questionnaires **(T4)**: 6. E: “*Please work on the questionnaires on the PC. After that, you can rest. I will come back in 15 min.“* |
|  | 1. E goes via room 290 to room 210, waits until stopwatch shows 25min |
| T_5_ +45min | 1. E goes to room 211 2. E sets 7.marker for HR(V) measurement **(T6HR(V))** 3. Salvia sample (Pb 1) **(T5)** 4. Instruction (I) ist the same as in T_0_ 5. Psychological questionnaires **(T5)**: 6. E: “*Please work on the questionnaires on the PC. After that, you can rest. I will come back in 20 min.“* |
| T_5_ +45min | 1. goes to room 212 2. E sets 7.marker for HR(V) measurement **(T6HR(V))** 3. Salvia sample (Pb 2) **(T5)**   Instruction (I) ist the same as in T_0_   1. Psychological questionnaires **(T5)**: 2. E: “*Please work on the questionnaires on the PC. After that, you can rest. I will come back in 20 min.“* |
| T_5_ +45min | 1. E goes from room 213. 2. E sets 7.marker for HR(V) measurement **(T6HR(V))** 3. Salvia sample (Pb 3) **(T5)**   Instruction (I) ist the same as in T_0_   1. Psychological questionnaires **(T5)**: 2. E: “*Please work on the questionnaires on the PC. After that, you can rest. I will come back in 20 min.“* |
|  | 1. E goes via 290 to room 210, waits until stopwatch shows 45min. (VL stops stopwatch and sets it to 0). |
| T_6_ +65min | 1. E goes to room 211 2. E sets 8.marker for HR(V) measurement **(T7HR(V))** |
|  | 1. Salvia sample (Pb 1) **(T6)**   Instruction (I) ist the same as in T_0_   1. Psychological questionnaires **(T6)**: 2. E: “*Please work on the questionnaires on the PC.”*   (Without BSSS)   1. E: "*Please take your vest off after this. I will come back and knock in a moment."* |
| T_6_ +65min | 1. E goes back to room 212 2. E sets 8.marker for HR(V) measurement **(T7HR(V))** 3. Salvia sample (Pb 2) **(T6)**   Instruction (I) ist the same as in T_0_   1. Psychological questionnaires **(T4)**: 2. E: “*Please work on the questionnaires on the PC.”*   (Including BSSS: Appendix H).   1. E: "*Please take your vest off after this. I will come back and knock in a moment."* |
| T_6_ + 65min | 1. E goes to room 213 2. E sets 8.marker for HR(V) measurement **(T7HR(V))** 3. Salvia sample (Pb 3) **(T6)**   Instruction (I) ist the same as in T_0_   1. Psychological questionnaires **(T6)**: 2. E: “*Please work on the questionnaires on the PC.*   (Without BSSS)   1. E: *"Please take the vest off afterwards. I'll be waiting behind the door. Please let me know as soon as you are finished.“*   *Note: If Pb 3 had a Cof2 with him or her, he or she also receives the BSSS |
|  | 1. E goes to room 211: "*The study is now over. We will now go back to the test room together with the other participants. There you will receive your payment. Please go to the test room* |
|  | 1. E goes to room 212.   E: "*The study is now over. We will now go back to the test room together with the other participants. There you will receive your payment. Please go to the test room*   1. E goest to room 213.   E: "*The study is now over. We will now go back to the test room together with the other participants. There you will receive your payment. Please go to the test room* |
|  | 1. E notes end time of study on her tablet |
| Duration 5-10min | **Debriefing** |
| +66min | E conducts debriefing (Appendix I), answers questions as needed. |
| +71-76min | 1. E pays pb individually 2. Pb confirms receipt of payment with signature on list of confirmation of payment. 3. Pb is dismissed and leaves the laboratory. |
| **Organizational matters after the study** | |
|  | When all Pb have left the lab:   1. Turn off cameras and microphones (if not already done). 2. Collect salicaps, check labeling and freeze. 3. Save questionnaires of each Pb, turn off PC/tablets, clean up. 4. Save HR(-V) measurements, turn off VL's tablet, clean up. 5. Disinfect pulse belts, tidy up 6. Reset rooms if necessary 7. Switch off lights, close doors |
