# Supplementary material for: Social Support as a Stress Buffer or Stress Amplifier and the Moderating Role of Implicit Motives: Protocol for a Randomized Study
Source: JMIR Res Protoc. 2022 Aug 9;11(8):e39509. doi: 10.2196/39509 (PMC9399871; doi:10.2196/39509)
Supplement: Multimedia Appendix 4 [file resprot_v11i8e39509_app4.docx]

Daily screening questionnaire

**Thank you for agreeing to participate in our study.**

In the following, we will ask you to answer some questions about yourself. Your data will of course be kept **strictly confidential** and **anonymous**!

Please generate your personal participant code here. It is very important that you generate it according to the following rules so that we can process your data anonymously.

To generate your code please enter: Example

the first letter of your mother's first name, A (Astrid)

the last letter of your father's first name, T (Helmut)

your month of birth as a number, 04 (April)

and the first letter of your first name. S (Simon)

Generated example code: AT04S

**1. How old are you?**

_ _ years

**2. Gender:**

women  men  diverse

**3**. **How tall are you (please provide whole numbers, no ranges of numbers)?**

_ _ _ cm

**4. How tall are you (please provide whole numbers, no ranges of numbers)?**

_ _ _ kilograms

**5. How long have you been awake today?**

_ _ _ hours

**6. Do you have a job (including voluntary and part-time jobs)?**

yes  no

**If no, please continue with question 7**

**6.1. If yes: Please state the job title.**

**7. Are you studying?**

yes  no

**If no, please continue with question 7.**

**6.1. If yes: Please name your major field of study.**

**8. Do you suffer from one or more (chronic) physical diseases (e.g. cardiovascular complaints, diabetes, neurological diseases, skin diseases, circulatory disorders, immune system disorders, endocrine diseases, etc.)?**

yes  no

**If no, please continue with question 9.**

**8.1. If yes: Which chronic disease(s)?**

**9. Do you suffer from allergies?**

yes  no

**If no, please continue with question 10.**

**9.1. If yes: Which allergies do you suffer from? Please name them briefly.**

**10. In the last three months, have you been treated in a hospital (operations, acute care) or had an operation in the oral cavity by a dentist / surgeon?**

yes  no

**If no, please continue with question 11.**

**10.1. If yes: How many months and days ago did this/these procedure(s) take place and what was it/they were (please indicate whole numbers, no number ranges)?**

Intervention____________________ took place _ _ months and_ _ days ago.

Intervention____________________ took place _ _ months and_ _ days ago.

Intervention____________________ took place _ _ months and_ _ days ago.

Intervention____________________ took place _ _ months and_ _ days ago.

**11. In the last year, have you experienced one or more massive changes in your life circumstances (e.g., moving, new job, marriage, divorce, birth of a child, death of a close friend or relative, etc.)?**

yes  no

**If no, please continue with question 12.**

**11.1. If yes: What were the changes? Please describe them briefly.**

**12. Do you take - besides hormonal contraceptives - another hormone preparation (e.g. thyroid hormones, general hormone substitution etc.)?**

yes  no

**If no, please continue with question 13.**

**12.1. If yes: Please explain in more detail (product name):**

**13. Do you take psychotropic drugs?**

yes  no

**If no, please continue with 14.**

**13.1. If yes: Which psychotropic drug(s) are you taking (product name(s)):**

**14. Do you regularly take medications that are not psychotropic or hormonal drugs?**

yes  no

**If no, please continue with 15.**

**14.1. If yes: What is the medication(s) (product name(s))?**

**15. Have you smoked regularly in your life for a period of more than 6 months? By regular, we mean at least 1 pack of cigarettes per month.**

yes  no

**If no, please continue with 16.**

**15.1. If yes: Do you currently smoke, even if only occasionally?**

yes  no

**16. Do you consume caffeine?**

yes  no

**If no, please continue with 17.**

**16.1. If yes: In what form do you consume caffeine and how often (please give whole numbers, no number ranges)?**

_ _ cups of coffee per day or _ _ _ cups of coffee per week.

_ _ cups of caffeinated tea per day or _ _ _ cups of caffeinated tea per week.

_ _ units of 0.5 liters of cola per day or_ _ _ units of 0.5 liters of cola per week.

_ _ units 0.5 liters of energy drink per day or_ _ _ units 0.5 liters of energy drink per week.

_ _ units 0.5 liters of iced tea per day or_ _ _ units 0.5 liters of iced tea per week..

_ _ caffeine tablets per day or_ _ _ caffeine tablets per week..

_ _ caffeine in a combined preparation (e.g. to combat flu symptoms) per day or_ _ _ caffeine in a combined preparation per week

**17. How often do you drink alcohol?**

never

about 1 time per month

2 - 4 times a month

2 - 3 times a week

4 times a week or more

**18. Do you regularly use narcotics ("drugs")?**

yes no

**If no, please continue with 19.**

**18.1. If yes: Which narcotic(s) are you using?**

**20. Do you regularly perform meditation, mindfulness or relaxation exercises?**

yes no

21. Did you perform any strenuous exercise (where you broke a sweat) 24 hours prior to this lab appointment?

yes no

**22. Did you perform any moderate activity (e.g., bicycling, walking) 2 hours prior to this lab appointment?**

yes no

**23. Did you perform any strenuous activity (in which you sweated profusely) 2 hours prior to this lab appointment (e.g., weight training, intense jogging)?**

yes no

24. How long has it been since your last large meal?

_ _ hours

**For female participants:**

**25. Do you have a menstrual period?**

yes  no

**If no, please continue with 26.**

**If yes, please continue with question 25.1.**

**25.1. When was the start of your last menstrual period (or period) (please give whole numbers, no number ranges)?**

_ _ days ago.

**25.2. Do you have your menstrual bleeding (or period) at regular intervals (are the time intervals between the start of your menstrual bleeding the same)?**

yes  no

**25.3. How long does your menstrual cycle usually last?**

less then 23 days

23-25 days

26-28 days

29-31 days

32-34 days

35-37 days

longer than 37 days

**25.4. How long does your menstruation (or period) usually last?**

1–2 days

3–4 days

5–6 days

7–8 days

more than 8 days

**26. Do you take a "birth control pill" for hormonal contraception?**

yes  no

**If no, please continue with 27.**

**26.1. If yes: Please explain in more detail (product name):**

**27. Do you use a hormonal contraceptive other than the "birth control pill" (e.g., vaginal ring, contraceptive patches and sticks, hormonal IUD, depot preparations (such as the three-month injection)?**

no  yes

**28. Is there currently a pregnancy?**

yes  no

**29. If you are already a mother, are you currently breastfeeding?**

yes  no
