# Supplementary material for: Social Support as a Stress Buffer or Stress Amplifier and the Moderating Role of Implicit Motives: Protocol for a Randomized Study
Source: JMIR Res Protoc. 2022 Aug 9;11(8):e39509. doi: 10.2196/39509 (PMC9399871; doi:10.2196/39509)
Supplement: Multimedia Appendix 5 [file resprot_v11i8e39509_app5.docx]

**Instructions for participants**

**Instruction Job-Interview**

Imagine that you are about to have an interview for a job of your choice. You can decide for yourself which job that is. Think of a job that you find very attractive. You are going to present yourself in front of a two-member panel that is going to decide about your employment. There will be other applicants in the room with you.

You will now have 10 minutes to prepare for a three-minute interview. You may take notes during the preparation time. However, you are not allowed to use these notes during the interview.

You should focus primarily on your personal qualities, i.e., list any positive personality traits that distinguish you from other applicants and qualify you for this position. You should not elaborate so much on your knowledge or professional qualifications; assume that all documents relating to your educational background, such as your resume, are already available to the panel.

The entire interview will be recorded by video and microphone for later analysis. Therefore, please speak loudly and clearly. Please also pay attention to facial expressions, gestures and language. The panel is trained in behavioral observation and will take notes on your appearance. They can ask you questions at any time, even if it is another person's turn to speak.

After the free speech, the panel will explain the next steps to you. You will always be called on at random and can take your turn again at any time. All of this will take 20 minutes together.

Your room number, which hangs on the wall, is also your participant number - you will always be called by this number.

You can take notes on the next page.

Do you have any questions? Please remember that they really want to get the job.
