# Supplementary material for: Social Support as a Stress Buffer or Stress Amplifier and the Moderating Role of Implicit Motives: Protocol for a Randomized Study
Source: JMIR Res Protoc. 2022 Aug 9;11(8):e39509. doi: 10.2196/39509 (PMC9399871; doi:10.2196/39509)
Supplement: Multimedia Appendix 6 [file resprot_v11i8e39509_app6.docx]

Social support instructions for the associate

General Instructions

- Confederate is female
- Confederate is generally friendly, enthusiastic, approachable and smiles at subject
- Confederate gives passive support during the first 5 minutes and only speaks when the subject addresses her.
- Confederate gives active support during the second 5 minutes: see standard response to minutes 5,7,9
- The social support response should appear as natural as possible: Confederate can deviate from standard response if the context requires it. Additional response options are listed in the table for other possible situations.
- If the subject explicitly does not want support (e.g., “I would like to keep working on my speech.”/ “Sorry, I need to focus on the task.”) 🡪 only the standardized support responses (after 5,7,9 minutes) are being offered
- No support through physical contact (e.g., patting on the back, hugging etc.)

- **Confederate should be able to express sentences of the initial situation and standard responses by heart and fluently!**

Initial situation:

Subject is sitting in the room. The investigator is with him/her. The confederate arrives.

Investigator: *„Hello XX.“*  [Turning to subject] *„This is XX. She is a student assistant at our department. She will be present during the 10-minute preparation stage, in case you need help”.*

Confederate [greeting Pb enthusiastically*]: „Hello, I am XX. I’ll keep an eye on the time. But I am also here for you if you need help. I still have some work to do on the computer but if I can help you in any way, please let me know. Just get in touch! “*

Investigator [Turning to confederate]: *„Thank you XX. “*  [Turning to participant*] „Start preparing for your speech now.“*

Investigator leaves the room.

Confederate [starts own stopwatch]: *„Time is running from now on! I wish you a lot of success. And as I said, just talk to me if you need help”*

The confederate sits down at the computer, behaves quietly and edits a table on the computer.

During the first five minutes of preparation, the confederate remains quiet and provides only passive support. She speaks only when the participant addresses her. The confederate is generally friendly, facing the participant and smiling. The 5 minutes of passive support is followed by 5 minutes of active social support with standardized support responses (minutes 5, 7, 9) for more details see G.2.

In each case, the confederate records the participant's response to the standardized support sentences. She also notes any special or unusual reactions of the participant, including the time. To make the social support situation appear as natural as possible, it is possible to deviate from the standard sentences in terms of time if the context requires it. Besides, the confederate receives further support sentences to be able to react to different situations (for more details see G.2 below). If a participant explicitly does not want support, the standardized support responses (after 5, 7, 9 min) are offered. At the end of the 10-min preparation period, the confederate asks for a saliva sample (T1, 0min) and announces the room change. A detailed procedure and formulation of support responses can be seen in the study protocol (see Appendix A).

Protocol Social Support

Subject-number: _____________ Date: ____________

Standard response

| Minute | Phrase | Reaction of Subject | Reactance? |
| --- | --- | --- | --- |
| 5 | *„So, how’s it going “ ^1^*  *Is there anything else I can help you with?” ^2^* |  |  |
| 7 | *„I’ve already participated in a study like this as well, so I know exactly how you feel. But in the end, it was not so bad.”^1^* |  |  |
| 9 | *„ Don’t worry, soon you’ll be through with it. And we are really grateful for your participation in our study. “ ^1^* |  |  |
| Shortly before subject is being sent in the TSST-G test room | *“I wish you a lot of success. You can do it.*” |  |  |

| Unique responses (if present) | Minute |
| --- | --- |
|  |  |

Other possible situations

| Min | Situation | Phrase | Reaction of Subject | Other phrases |
| --- | --- | --- | --- | --- |
|  | Subject seems nervous/ anxious/ tense | *„It is perfectly normal to be a little nervous.“*  *„Don’t worry, I’m sure you will do great!“* ^a^ |  |  |
|  | Subject has a question about the process of the TSST | Only repeat information from the information sheet. ^b^ |  |  |
|  | Subject has a question about the task | Explain the task once again ^b^  *„Try to think about why exactly you are qualified for the job! Have you maybe done an internship in that direction or worked in that field before?“ ^b^* |  |  |
|  | Subject is afraid of embarrassing himself/herself/ failing | *„It is completely normal to feel this way.“* ^a^  *„But in the end, it wasn’t so bad!!“ ^a^* |  |  |
|  | Subject does not like to speak in front of groups/ give talks | *„Yes, I know that. But once I’ve started, it often works out better than I had thought. “* ^a^  *„I can completely understand! But you’ve certainly given talks before, right? I bet they were always good “* ^a^ |  |  |
|  | Subject does not feel like doing the task/ is irritated | *„Yes, I can understand. But don’t worry, soon you’ll be through with it. And we are really grateful for your participation in our study!“* ^a^ |  |  |

Other possible situations – Table part 2

|  | Subject asks for hints | *„Sometimes it helps to breathe in deeply before starting.“* ^b^  *„It can also help to go over the talk once.* *If you want to practice your talk with me, we can do that.“ ^b^* |  |  |
| --- | --- | --- | --- | --- |
|  | Subject seems frustrated | „All good?“ ^a^  „Are you all right?“ ^a^  „Is there anything I can do to help?“ ^2^ |  |  |

emotional support = ^a^
informational support = ^b^
