# Supplementary material for: Social Support as a Stress Buffer or Stress Amplifier and the Moderating Role of Implicit Motives: Protocol for a Randomized Study
Source: JMIR Res Protoc. 2022 Aug 9;11(8):e39509. doi: 10.2196/39509 (PMC9399871; doi:10.2196/39509)
Supplement: Multimedia Appendix 7 [file resprot_v11i8e39509_app7.docx]

**Procedure for the panel**

Standardized TSST-G procedure for panel members with comments Interview & computational task.

**General Rules:**

- When meeting the participants for the first time, the panel does not show any reaction and only says "Good afternoon" coldly and without smiling if the participants greet them.
- If someone speaks to the participants, it is the active panel member (GM2).
- Panel member does not show any positive reaction to the participants, rather neutral and distant basic attitude
- Goal: to make the participants feel insecure and stressed. To achieve this, the participant can be deliberately upset with questions, allowed to speak without feedback, or interrupted spontaneously.

Participants take their places, VL helps if needed.

**1.1 Interview 3x3min**

**GM 2: starts stopwatch 1** (must run continuously)

GM 2: *"We will now begin the interviews. Once your number is called, please step forward to the mark. Your speech will be recorded by video camera. The camera will be aligned first and you will then be asked to begin your speech. Once all candidates have finished their speech, another task will follow."*

GM 2*: "Number X1 step forward and stand exactly at the mark."*

GM 1: Align camera & microphone. Sits down & signals GM 2 to proceed by nodding head.

GM 2: *"Please begin your presentation with your participant number and today's date. Begin now!"*

GM 1 starts stopwatch 2 (places it between himself and GM 2, signals GM 2 at 2:45min).

If participant has trouble starting (optional) prompt GM 2: *"Briefly say what you are applying for."*

*Initially let participants speak freely*. **Approximately one interruption** every minute. *Question 4 only in case of emergency (see interruption sets).*

**Interruption sets**

| ***Participant 1*** | ***Participant 2*** | ***Participant 3*** |
| --- | --- | --- |
| 1. *How do you work under time pressure?* 2. *Thank you, we are not interested in that so much. Please address your greatest strengths.* 3. *What leadership qualities do you possess?* 4. *What are your occupational goals?* | 1. *How do you deal with critique?* 2. *That doesn't really fit now, thank you. Please address your problem solving strengths.* 3. *Why do you consider yourself more suitable than other applicants? What makes you stand out?* 4. *What do you think about teamwork?* | 1. *Why do you think you are specially qualified for this task?* 2. *Thank you, but this is less relevant. Please address your strengths in organizational and planning processes.* 3. *How many hours of extra work are you willing to do each week?* 4. *What do your family and friends appreciate most about you?* |

**If the participant stops talking:** look at him sideways and take notes.

If the participant stops talking before the 3 minutes are up, point out that they still have some time:

Short pause, then GM 2: ...

(0) *"You still have time, please continue."*

**Emergency questions:**

(1) "*How do you feel about job interviews?"*

(2) "*How eloquent do you think you are?"*

(3) *"Please complete the sentence, I am the best at ..."*

(4) *"Are you vain?"*

(5) *"What situations are embarrassing to you?"*

(6) *"To what extent do you find it possible to combine a successful professional career with a fulfilling family life?"*

(7) *"What actions are you taking to still be working for us in 5 years?"*

**After 3min of the participant's speech:**

Interrupt the participant ideally in mid-sentence and move on to the next participant.

GM 2: *"Thank you, that's enough."*

GM 1: Notes speaking time from stopwatch 2, sets it back to 0.

GM 2: "*Number X2 step forward and stand exactly at the mark."*

GM 1: Aligns camera & microphone. Sits down & signals GM2 to proceed by nodding head.

GM 2: *"Please begin your presentation with your participant number and today's date. Begin now!"*

GM 1 starts stopwatch 2 (places it between himself and GM 2, signals GM 2 to time at 2:45min).

Same procedure as for participant 1.

**After 3min of the participant's speaking time:**

Interrupt participant ideally in middle of sentence and move on to next participant.

GM 2: "*Thank you, that's enough."*

GM 1: Notes speaking time from stopwatch 2, sets it back to 0.

GM 2: *"Number X3 step forward and stand exactly at the mark."*

GM 1: Aligns camera & microphone. Sits down & signals GM 2 to proceed via head nod.

GM 2: *"Please begin your presentation with your participant number and today's date. Begin now!"*

GM 1 starts stopwatch 2 (places it between himself and GM 2, signals GM 2 to time at 2:45min).

**After 3min of speaking time of the third participant: (time 11/12min)**

Interruption of the participant ideally in the middle of the sentence

GM 2: *"Thank you, that's enough."*

*(Instruction for saliva delivery) "Take the tube that you will find on the right next to you on the partition in a blue bag. Collect the saliva at the front of your mouth and squeeze it through the straw. Try not to blow through the straw while doing this! When you have collected enough saliva, please close the small tube carefully (until it "clicks") and put the saliva sample back into the appropriate bag.* ***It is enough saliva when the tube is a little more than half full!*** *You can check in between how much is already in the container and then continue to dispense saliva until the required amount is reached. Also, please be careful to avoid bubbles in the sample."*

GM 1: Notes the speaking time of stopwatch 2, the latter resets to 0

**1.2 Filling out the psychological questionnaire**

GM2: *“Fill out the questionnaire that is on the clipboard.”*

**2. Arithmetic task 3x3x30sec**

Each Participant gets 3 times for 30 seconds. Participant must always start with a new starting number and count backwards in steps of 17. See below for order (checklist).

At the end of the interview of all 3 persons (time 12min) & saliva delivery transition to the arithmetic task: GM2 says indifferent and neutral (memorize!):

GM2: "*We now come to the second task. This is a math task. We will call on you again by your numbers. Then you step up to the marker, and count backwards in increments of 17 from the number you were told. You will always be called at random and can take your turn again at any time."*

GM2: *"Number X step forward."*

GM2: "*Please count backwards in increments of 17 from the number* ***4878****. Do this as quickly and as error-free as you can.*

*If you make a mistake, we will bring it to your attention and you will then have to start over at* ***4878****. Please start now."*

GM 1 starts stopwatch 1 after 25sec Note to GM 2.

GM2 checks numbers

After 30sec: GM2: *"Thank you, that's enough."*

GM 1 sets stopwatch 1 to 0.

**Checkliste**

**7**

**6**

| 4878 | 4861 | 4844 | 4827 | 4810 | 4793 | 4776 | 4759 | 4742 | 4725 | 4708 |
| --- | --- | --- | --- | --- | --- | --- | --- | --- | --- | --- |
| 4691 | 4674 | 4657 | 4640 | 4623 | 4606 | 4589 | 4572 | 4555 | 4538 | 4521 |
| 4504  **1** | 4487 | 4470 | 4453 | 4436 | 4419 | 4402 | 4385 | 4368  **5** | 4351 | 4334 |
| 4317 | 4300 | 4283 | 4266 | 4249  **2** | 4232 | 4215 | 4198 | 4181 | 4164 | 4147 |
| 4130 | 4113 | 4096 | 4079 | 4062 | 4045 | 4028  **9** | 4011 | 3994 | 3977 | 3960 |
| 3943 | 3926 | 3909 | 3892 | 3875 | 3858 | 3841 | 3824 | 3807 | 3790 | 3773 |
| 3756 | 3739 | 3722 | 3705 | 3688 | 3671 | 3654 | 3637 | 3620 | 3603  **3** | 3586 |
| 3569 | 3552 | 3535 | 3518 | 3501 | 3484 | 3467 | 3450 | 3433 | 3416 | 3399 |
| 3382 | 3365 | 3348 | 3331 | 3314 | 3297 | 3280 | 3263 | 3246 | 3229 | 3212 |
| 3195 | 3178 | 3161  **4** | 3144 | 3127 | 3110 | 3195 | 3178 | 3161 | 3144 | 3127 |
| 3110 | 3093 | 3076 | 3059 | 3042 | 3025 | 3008  **8** | 2991 | 2974 | 2957 | 2940 |

GM2: *"Number X step forward."*

GM2: *"Please count backwards in increments of 17 from the number* ***4623****. Do this as quickly and as error-free as you can.*

*If you make a mistake, we will bring it to your attention and you will then have to start over at* ***4623****. Please start now."*

GM 1 starts stopwatch 1 after 25sec Note to GM 2.

GM2 checks numbers

After 30sec: GM2: "*Thank you, that's enough."*

GM 1 sets stopwatch 1 to 0.

GM2: *"Number X step forward."*

GM2: *"Please count backwards in increments of 17 from the number* ***3977****. Do this as quickly and as error-free as you can.*

*If you make a mistake, we will bring it to your attention and you will then have to start over at* ***3977****. Please start now."*

GM 1 starts stopwatch 1 after 25sec Note to GM2.

GM2 checks numbers

After 30sec: GM2: *"Thank you, that's enough."*

GM 1 sets stopwatch 1 to 0.

Repeat procedure until (9) 4215

**If the participant makes a mistake:**

GM2: "*That was not correct. Please start over, XXXX (state starting number)."*

If too many correct answers or if they take too long:

GM2: *"Please try to calculate faster!"*

**Ignore all questions (except for starting number) from participants. In response to questions, "Please continue!"**

In case of emergency on questions: *"Questions will be answered later!"*

**After all 3 participants: (time 19/20min)**

GM2: *"Thank you, that's enough."*

(panel takes notes)

GM2: *(Instruction for saliva collection) "Take the tube that you will find on the left side of you on the partition in an orange bag. Collect the saliva in the front of your mouth area and squeeze it through the straw. Try not to blow through the straw while doing this! When you have collected enough saliva, please close the small tube carefully (until it "clicks") and put the saliva sample back into the appropriate bag. It is enough saliva when the tube is a little more than half full! You can check in between how much is already in the container and then continue to dispense saliva until the required amount is reached. Also, please be careful to avoid bubbles in the sample."*

VL steps forward, *"I will pick you up one by one. Please keep them standing until then."*

VL leads Pb individually to respective preparation rooms
