# Supplementary material for: Social Support as a Stress Buffer or Stress Amplifier and the Moderating Role of Implicit Motives: Protocol for a Randomized Study
Source: JMIR Res Protoc. 2022 Aug 9;11(8):e39509. doi: 10.2196/39509 (PMC9399871; doi:10.2196/39509)
Supplement: Multimedia Appendix 8 [file resprot_v11i8e39509_app8.docx]

**Manipulation check for social support**

Now please think about the student assistant. How did she behave towards you during the preparation stage for the job interview? Please mark with a cross where applicable.

|  | Not true | Barely true | Rather true | Exactly true |
| --- | --- | --- | --- | --- |
| Did the student assistant support you? |  |  |  |  |
| The student assistant showed me that she likes and accepts me. |  |  |  |  |
| The student assistant was there for me, when I needed her. |  |  |  |  |
| The student assistant cheered me up when I felt stressed. |  |  |  |  |
| The student assistant made me feel that I was making an important contribution. |  |  |  |  |
| The student assistant expressed concern for my wellbeing. |  |  |  |  |
| The student assistant made me feel that I could rely on her. |  |  |  |  |
| The student assistant helped me to find something positive in my situation. |  |  |  |  |
| The student assistant suggested an activity that could distract me a bit. |  |  |  |  |
| The student assistant encouraged me not to give up. |  |  |  |  |
| Did you experience the social support as being helpful? |  |  |  |  |
