# Supplementary material for: Social Support as a Stress Buffer or Stress Amplifier and the Moderating Role of Implicit Motives: Protocol for a Randomized Study
Source: JMIR Res Protoc. 2022 Aug 9;11(8):e39509. doi: 10.2196/39509 (PMC9399871; doi:10.2196/39509)
Supplement: Multimedia Appendix 9 [file resprot_v11i8e39509_app9.docx]

Debriefing form

I would now like to clarify for you what the job interview and the study were all about:

- With this study we intend to investigate the effects of social support and social motives on people’s stress experience.
- The goal of the job interview was to stress you. Because in order to investigate whether social support and social motives have effects on stress, we initially always need a stressed person. In today’s case, this was you.
- The online questionnaires before the study measured the social motives. The/A student assistant provided social support to a part of you.
- You first had to apply to a fictitious job, with no feedback from our side at all.
- The second task was mental arithmetic which of course was only used to stress you out.
- Basically, everything is set up in a way that it is impossible to perform perfectly.
- The persons on the committee were not professionals in behavioral observation and they were not trained for it.
- The person on the committee who spoke is also just a student and works in our team.
- The video recording and the microphone were additional elements of stress.
- In general, our job interview is structured in such a way that nobody can complete the tasks without being stressed. Of course, a normal job interview proceeds differently.
- The different cognitive tasks are used to investigate whether the typical effects of stress on cognitive performance can be influenced by social support and social motives.
- Say something positive (such as: “You handled the stress test well. You were very likable which is why it was particularly difficult for us/me to remain serious.”)
- Do you still have questions about the fictitious job interview or the study?
- We ask you not to pass on the contents of the study to other persons, to guarantee the same starting situation for all the participants.
- Thank you for taking the time to participate in the study.

**Remuneration & Acknowledgment of participation**
