# Supplementary material for: Social Support as a Stress Buffer or Stress Amplifier and the Moderating Role of Implicit Motives: Protocol for a Randomized Study
Source: JMIR Res Protoc. 2022 Aug 9;11(8):e39509. doi: 10.2196/39509 (PMC9399871; doi:10.2196/39509)
Supplement: Multimedia Appendix 10 [file resprot_v11i8e39509_app10.docx]

**Test battery of psychological questionnaires**

Dear Participant,

please answer the following questions truthfully, referring to your current situation. There is no "right" or "wrong".

At the moment I feel...

|  | not at all | not | partly | more like | very |
| --- | --- | --- | --- | --- | --- |
| good | 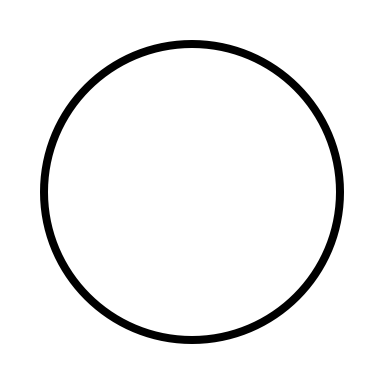 | 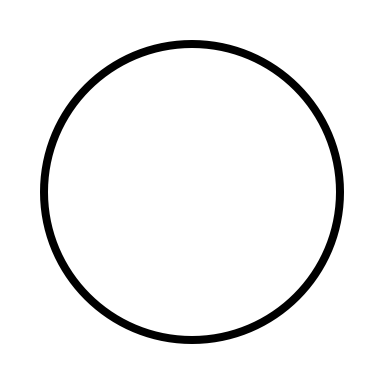 | 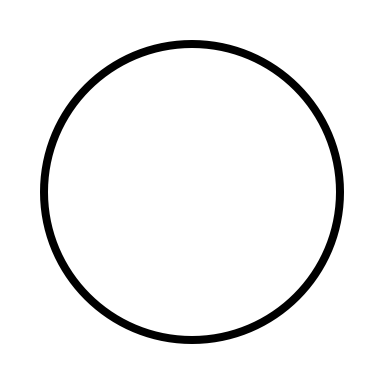 | 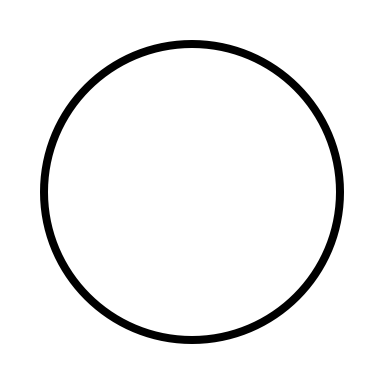 | 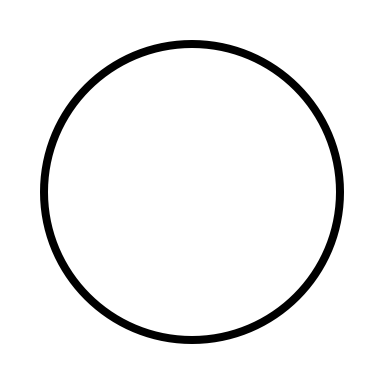 |
| bad | 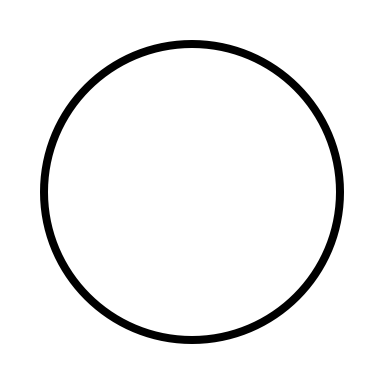 | 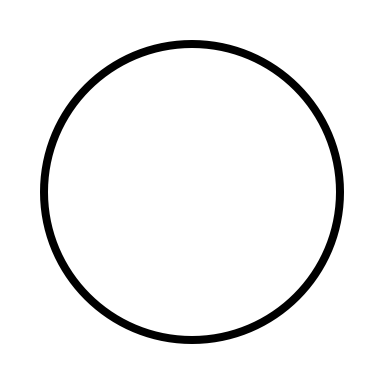 | 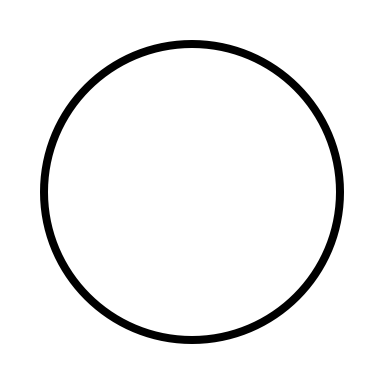 | 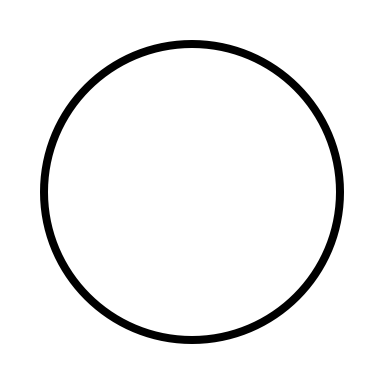 | 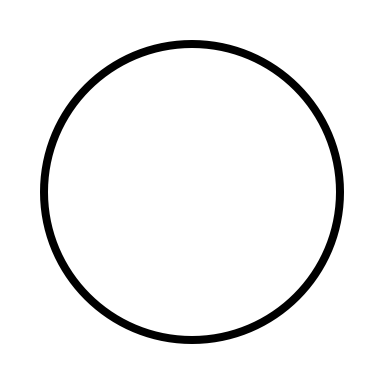 |
| fatique | 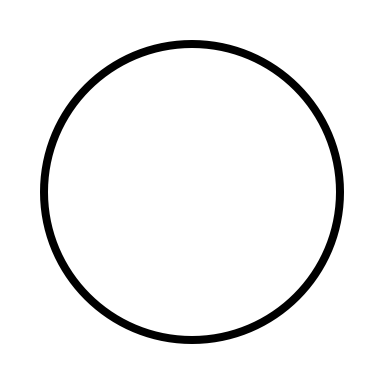 | 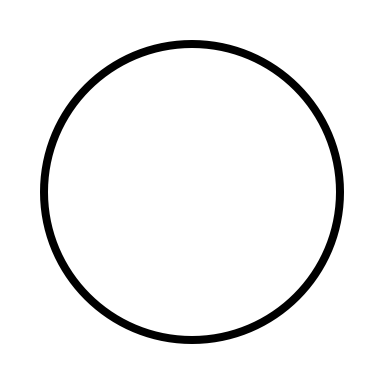 | 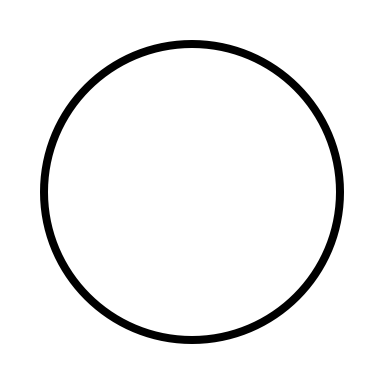 | 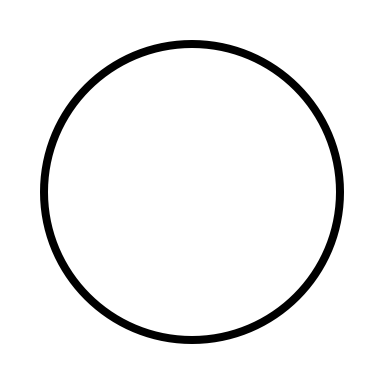 | 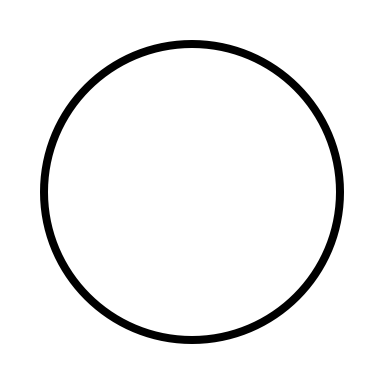 |
| alertness | 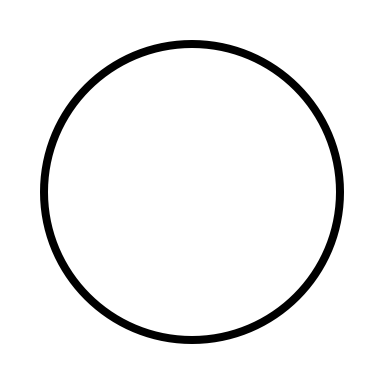 | 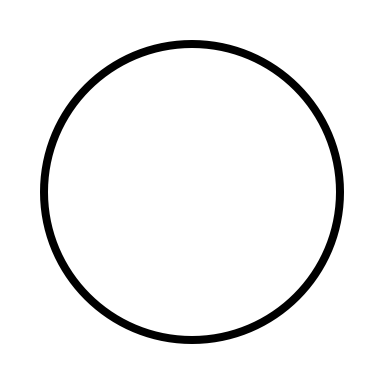 | 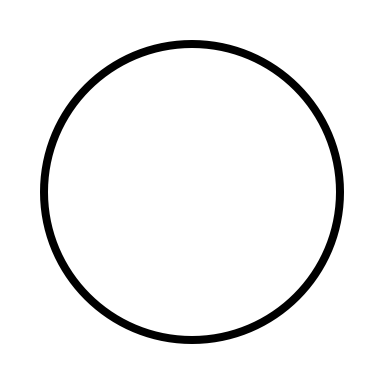 | 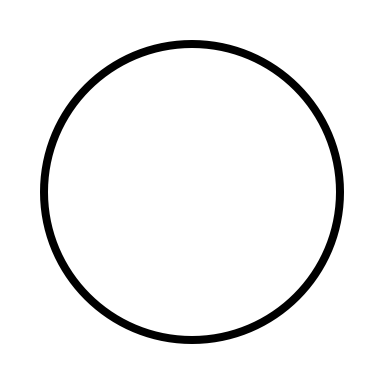 | 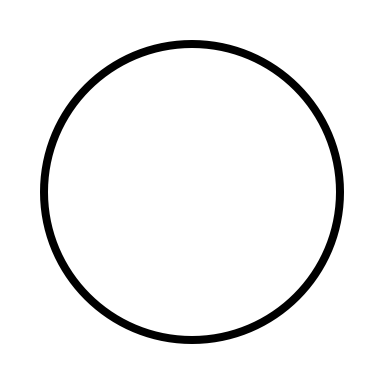 |
| restlessness | 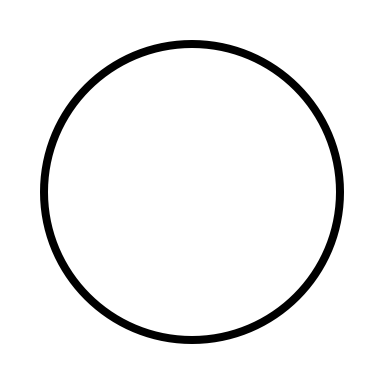 | 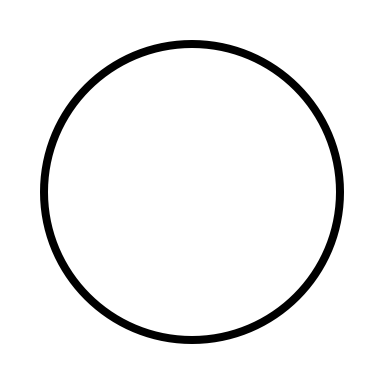 | 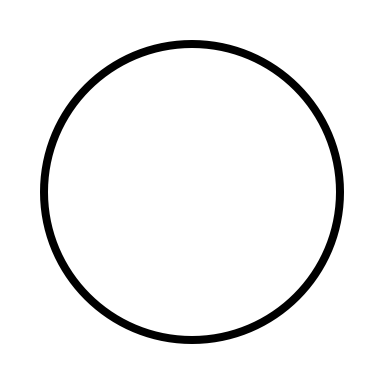 | 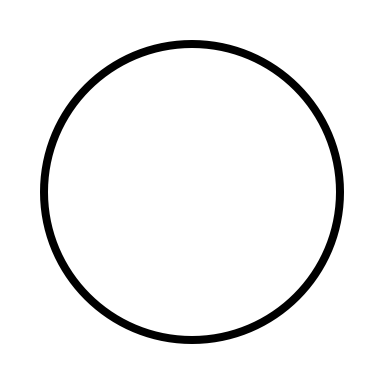 | 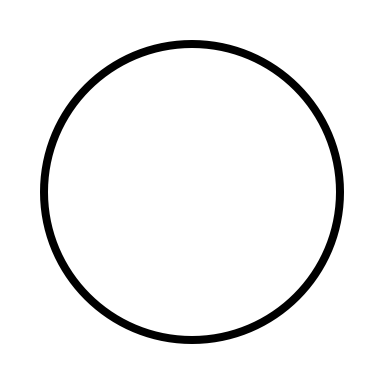 |
| relaxed | 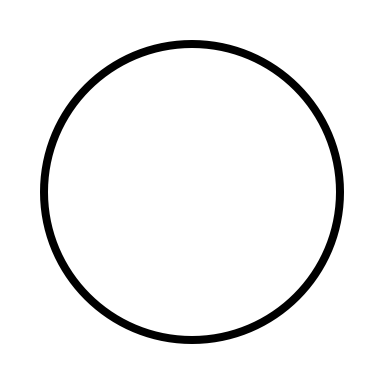 | 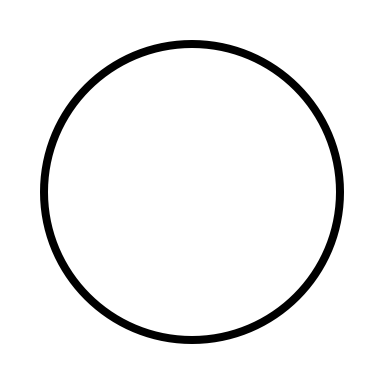 | 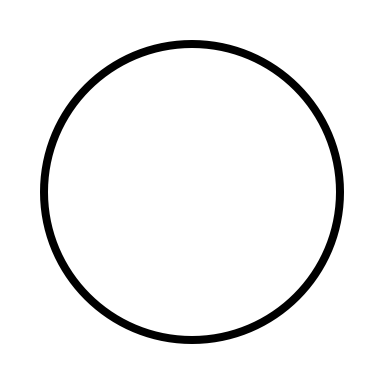 | 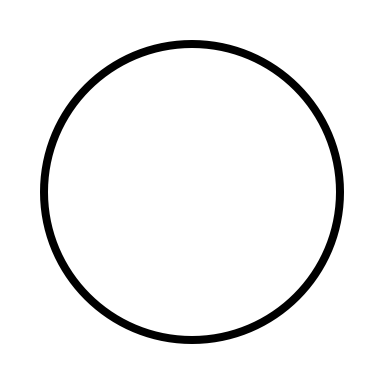 | 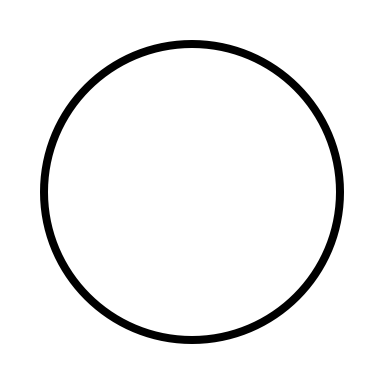 |

*Note.* Steyer, R., Schwenkmezger, P., Notz, P., & Eid, M. (1997). Der Mehrdimensionale Befindlichkeitsfragebogen MDBF [Multidimensional mood questionnaire]. *Göttingen, Germany: Hogrefe*.

Please indicate how you feel right now.

|  | 1  not at all | 2 | 3 | 4 | 5 | 6 | 7  very much |
| --- | --- | --- | --- | --- | --- | --- | --- |
| competent | 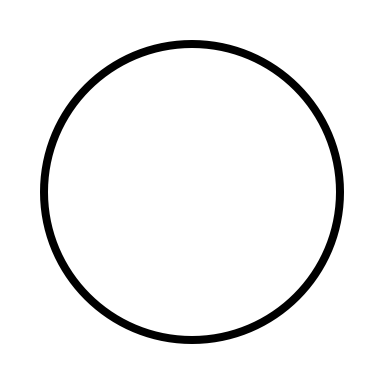 | 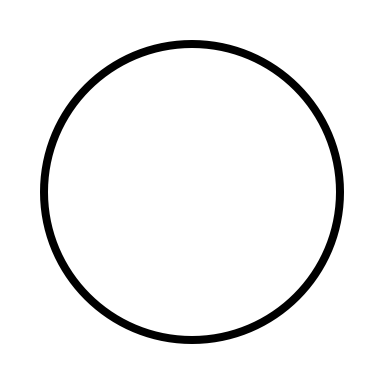 | 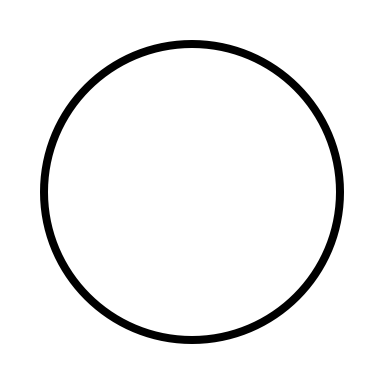 | 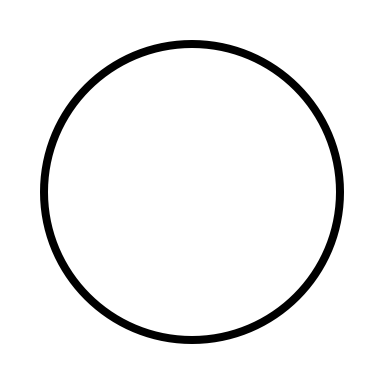 | 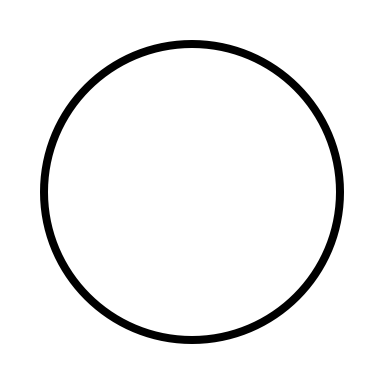 | 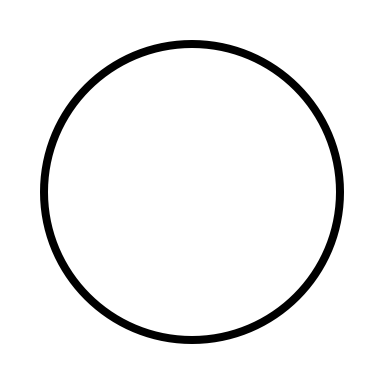 | 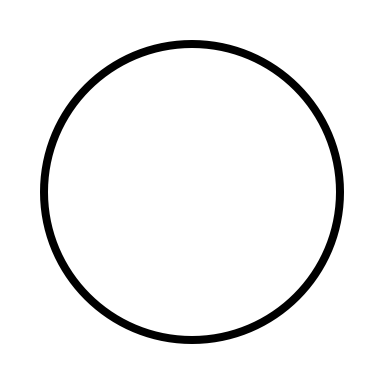 |
| socially integrated | 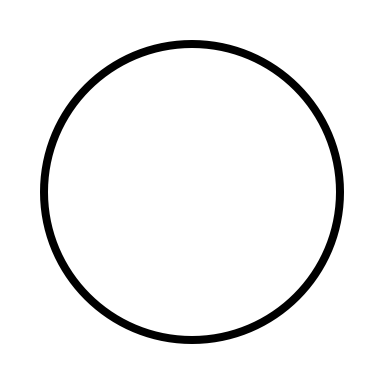 | 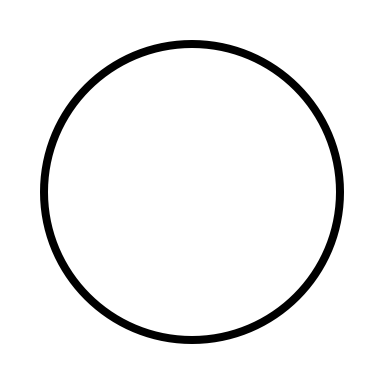 | 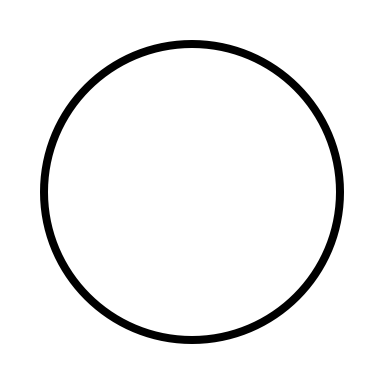 | 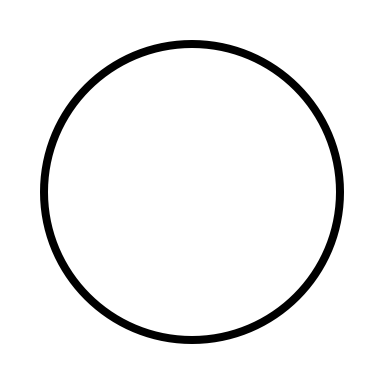 | 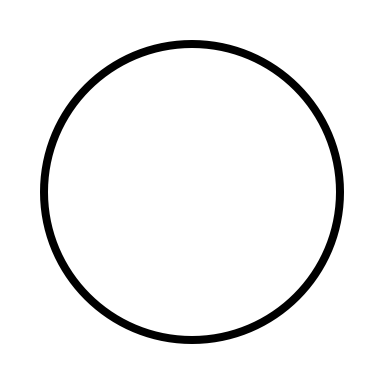 | 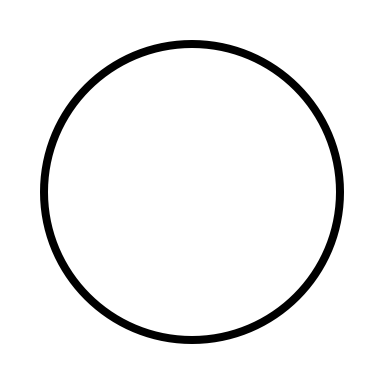 | 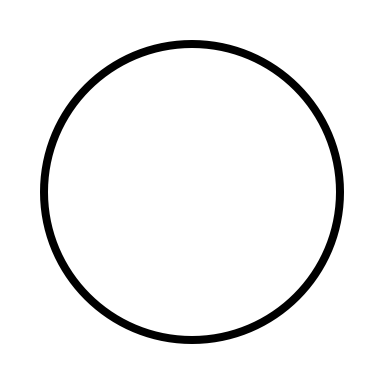 |
| strong | 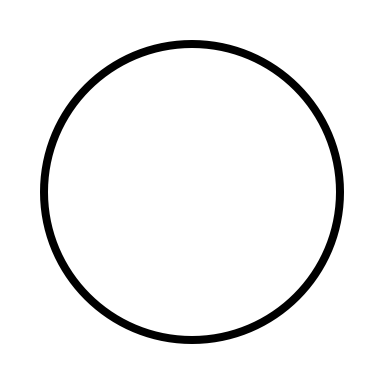 | 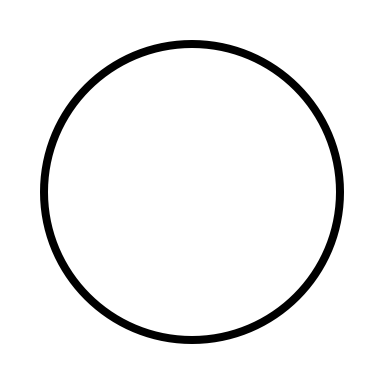 | 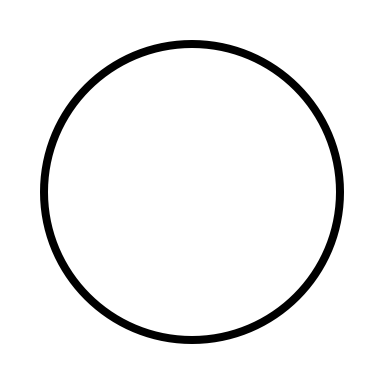 | 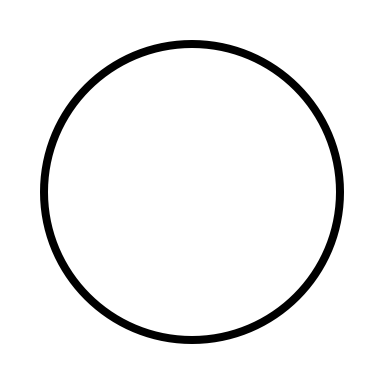 | 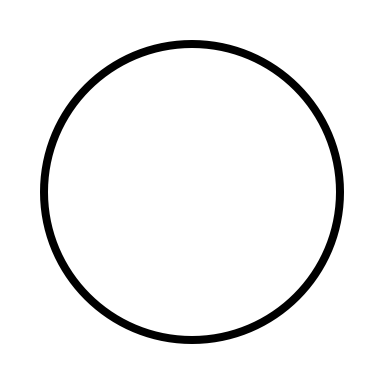 | 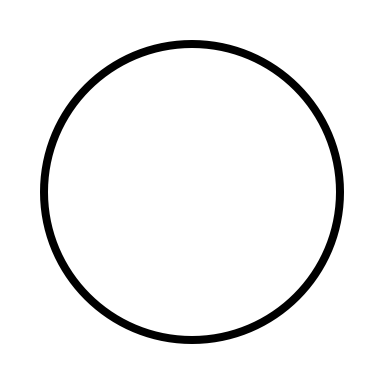 | 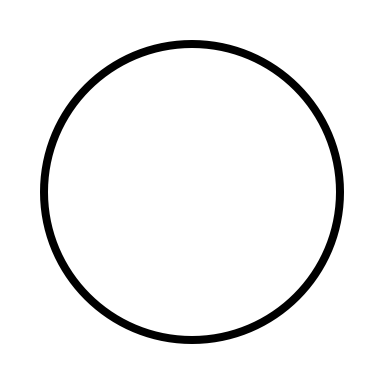 |
| self-determined | 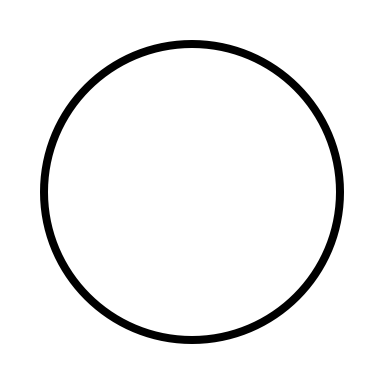 | 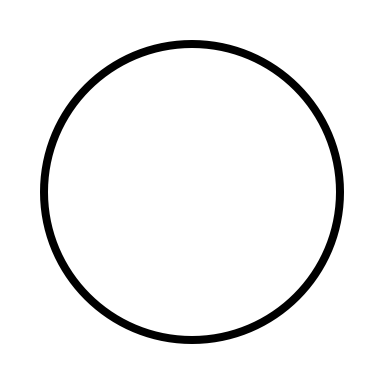 | 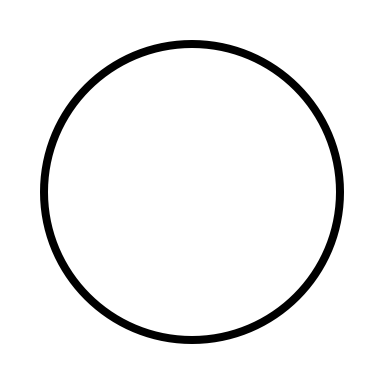 | 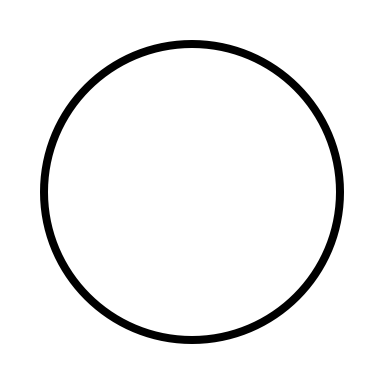 | 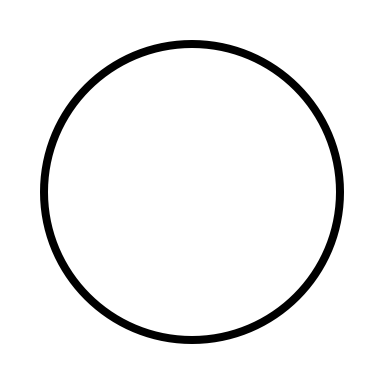 | 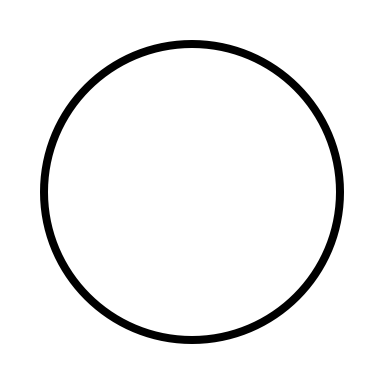 | 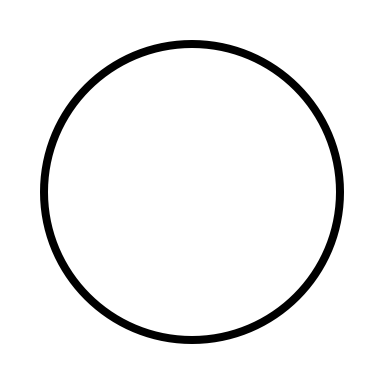 |
| calm | 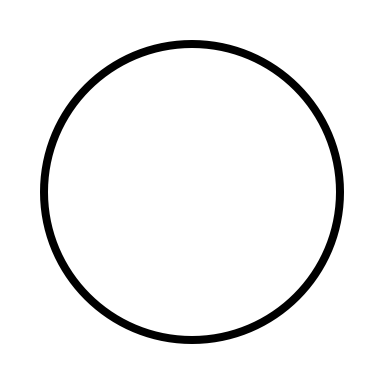 | 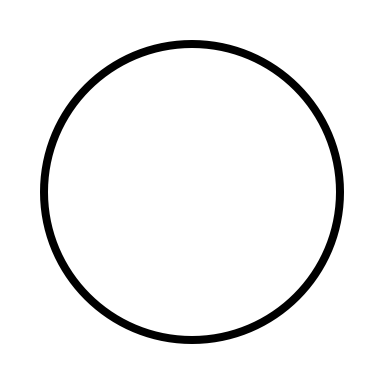 | 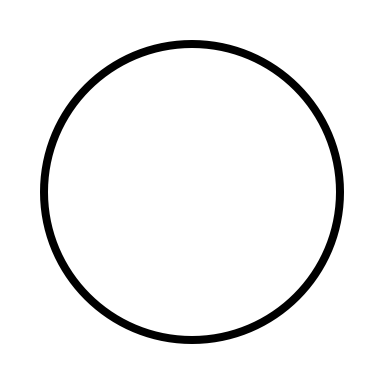 | 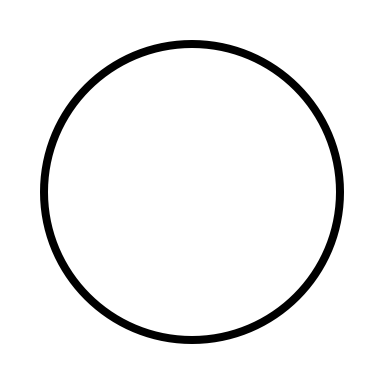 | 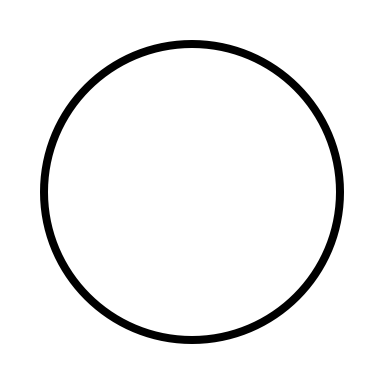 | 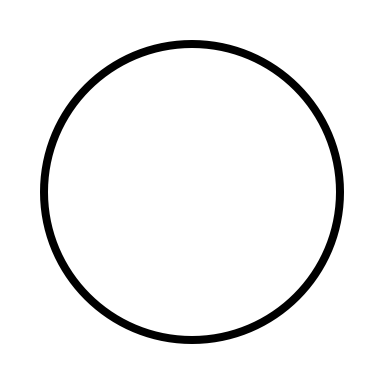 | 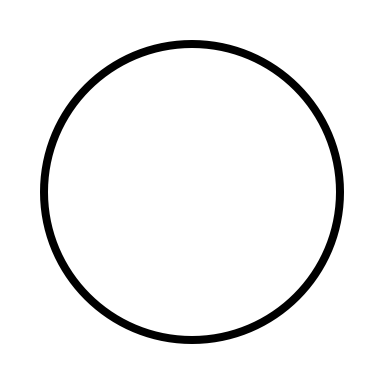 |
| excited | 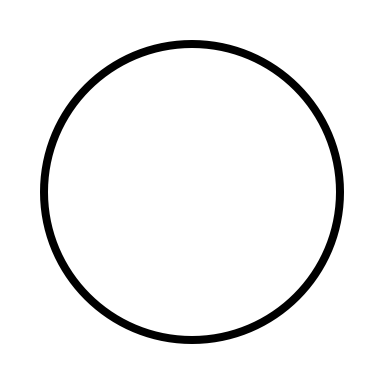 | 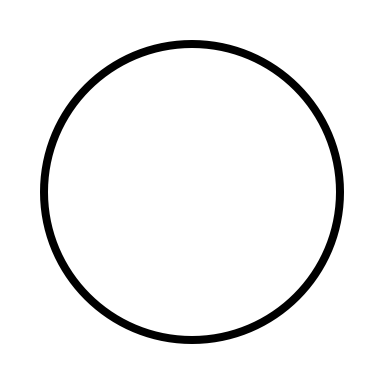 | 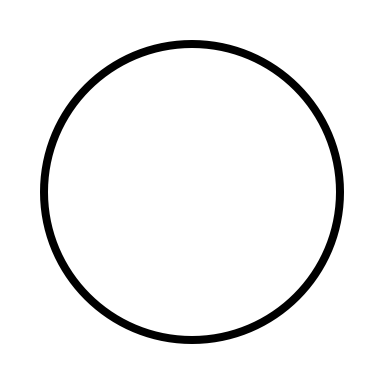 | 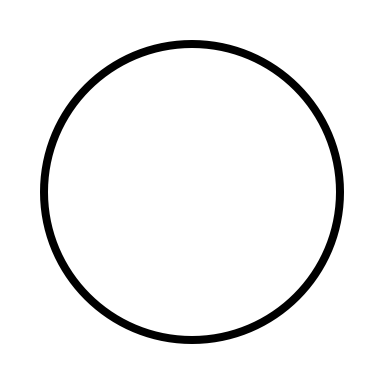 | 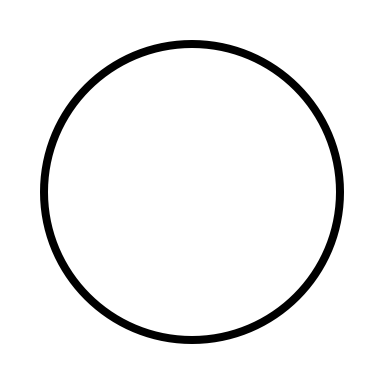 | 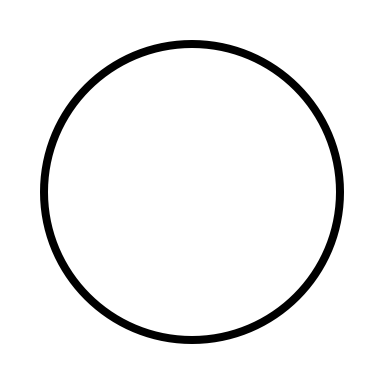 | 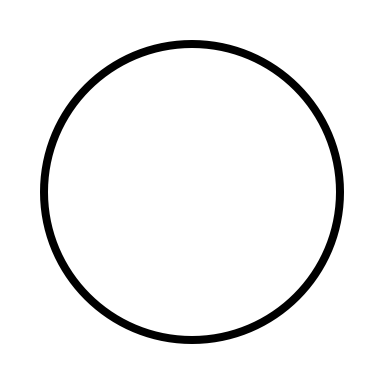 |
| enthusiastic | 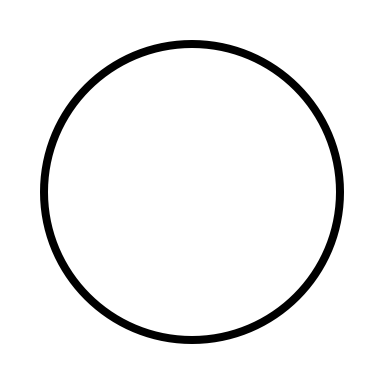 | 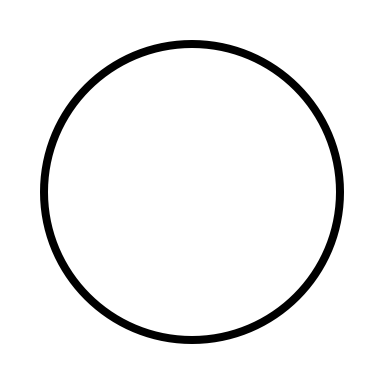 | 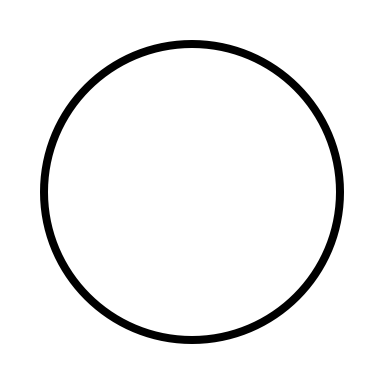 | 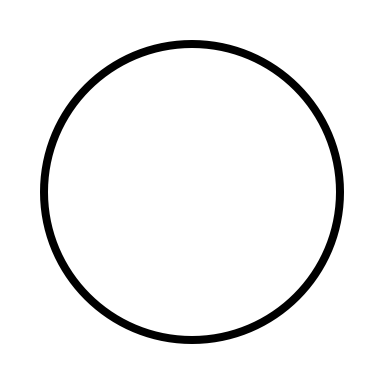 | 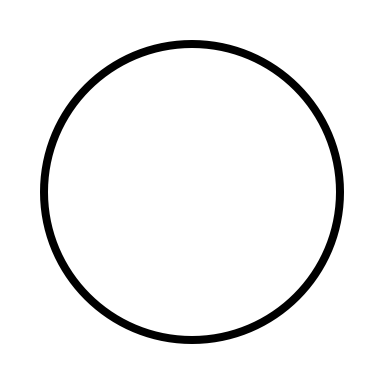 | 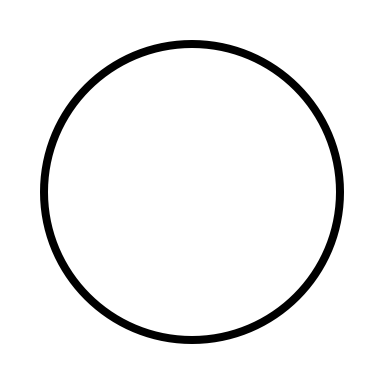 | 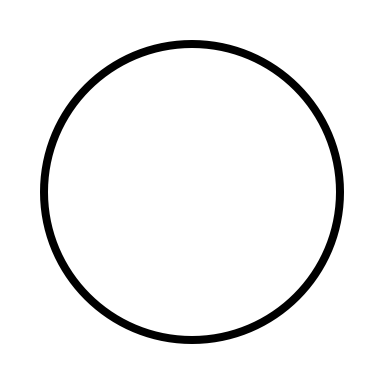 |

Please answer the following questions.

|  | no fear |  |  |  | great fear |
| --- | --- | --- | --- | --- | --- |
| How big do you think your fear is at the moment? | 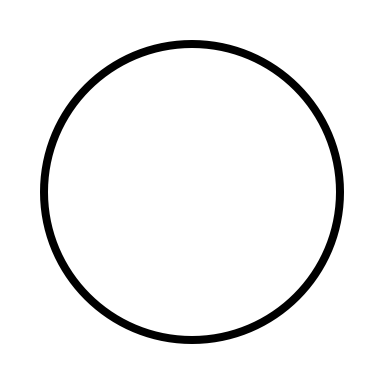 | 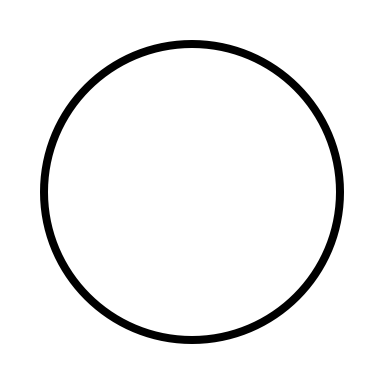 | 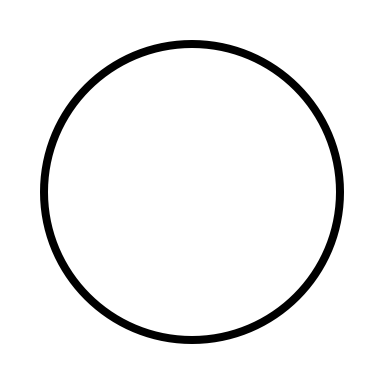 | 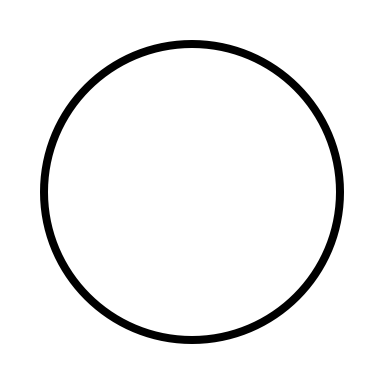 | 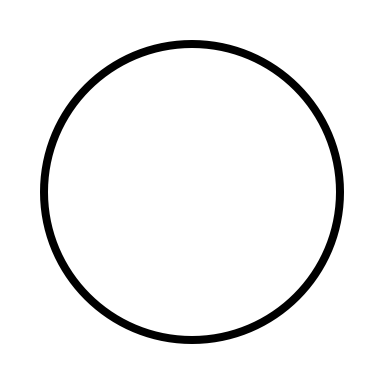 |
|  | not uncomfortable |  |  |  | very uncomfortable |
| How much do you feel physically uncomfortable right now? | 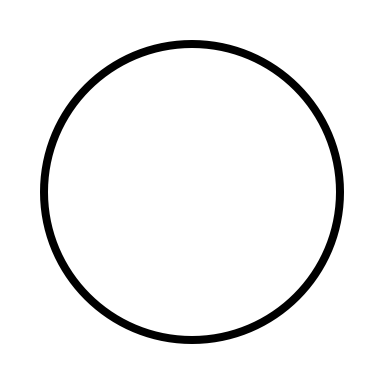 | 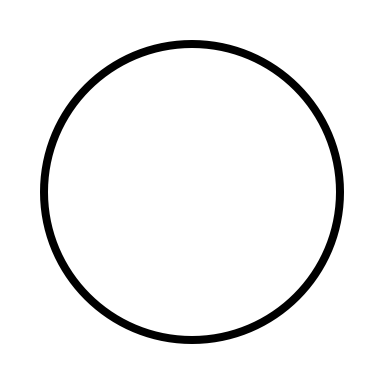 | 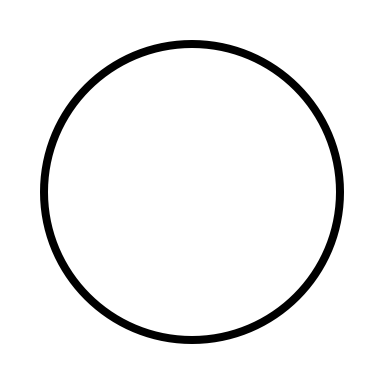 | 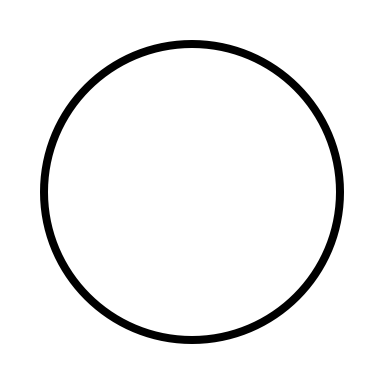 | 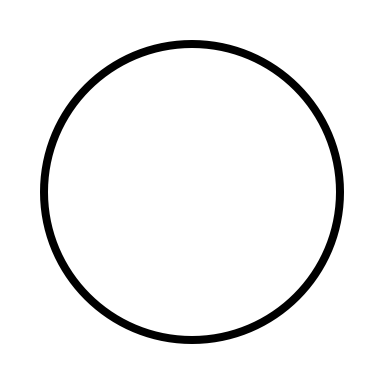 |
|  | not at al |  |  |  | very strong |
| How strong is your need to leave the situation? | 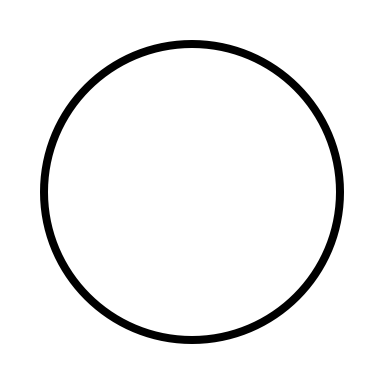 | 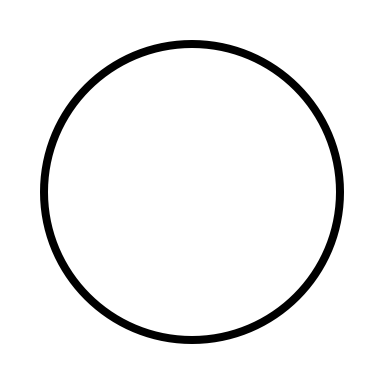 | 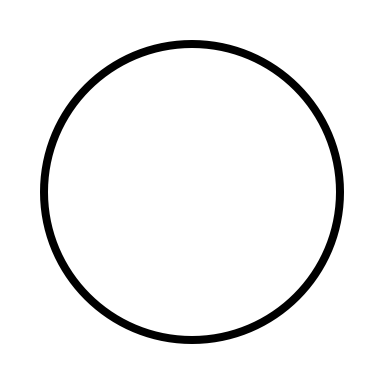 | 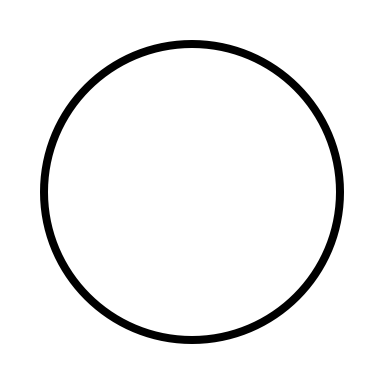 | 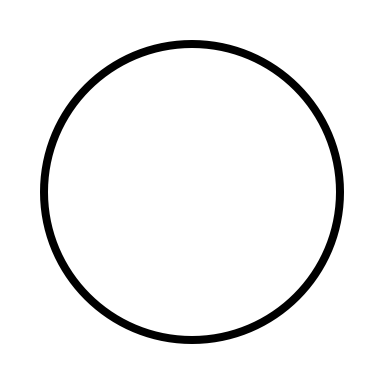 |
|  | not at al |  |  |  | very tense |
| How tense is your feeling right now? | 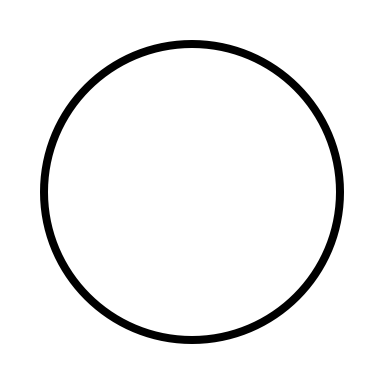 | 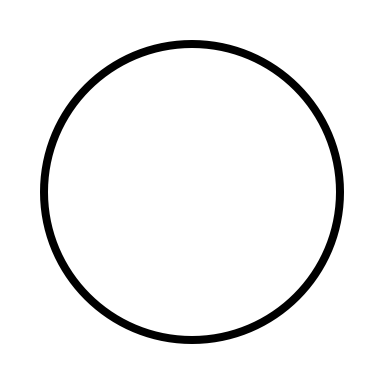 | 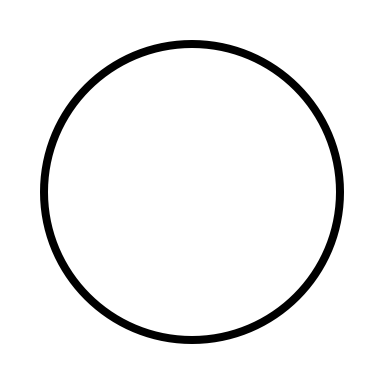 | 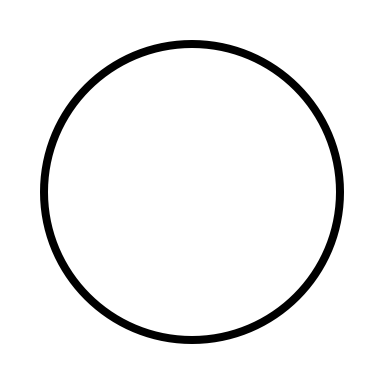 | 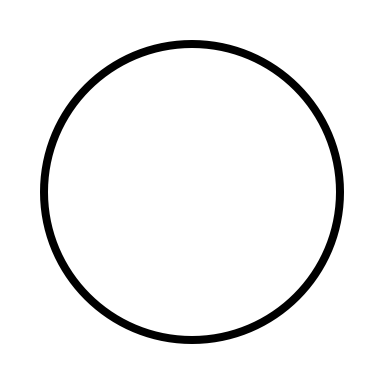 |
|  | not at al |  |  |  | very strong |
| How much are you in control of the situation? | 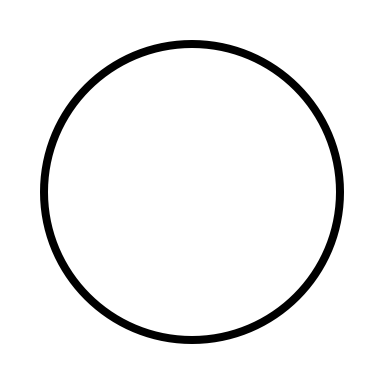 |  |  |  |  |
|  | not at al |  |  |  | very strong |
| How stressed do you feel? |  |  |  |  |  |

*Note.* Modified according to*:* Spielberger, C. D. (1970). Manual for the State-trait Anxietry, Inventory. *Consulting Psychologist*.
