# Supplementary material for: Social Support as a Stress Buffer or Stress Amplifier and the Moderating Role of Implicit Motives: Protocol for a Randomized Study
Source: JMIR Res Protoc. 2022 Aug 9;11(8):e39509. doi: 10.2196/39509 (PMC9399871; doi:10.2196/39509)
Supplement: Multimedia Appendix 11 [file resprot_v11i8e39509_app11.docx]

Instructions for the collection and processing of saliva samples

| **Preparation**  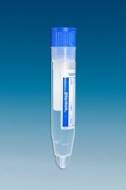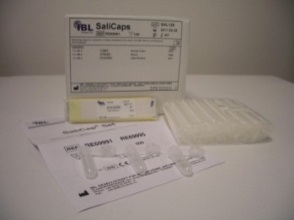 | **Prepare material**   - Prepare Salicaps in bags with a straw - Important: wear gloves; do not touch the inside of the container (especially the cover) |
| --- | --- |
| **Hygiene**  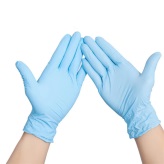 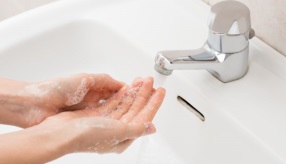 | **Important notes**   - Handle tubes only with latex gloves (also when gluing) - Wash your hands thoroughly after each contact with the tubes - In case of contact with saliva on the skin: disinfect hands - In case of contact with saliva on mucous membranes (mouth/nose etc.) or in wounds: consult a doctor - Only store samples in tightly closed bags or plastic boxes in the same refrigerator in which food is stored |
| **Collection**  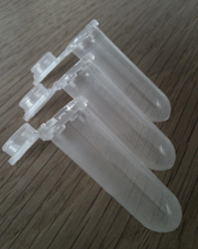 | **Saliva sampling with Salicaps**   - Fill Salicaps halfway with saliva using a straw, pressing the saliva through the tube with the tongue. Do not blow through the tube. - Collection should take exactly one minute (it is best to use a stopwatch) - Throw away straw and close Salicaps tightly |
| **Storage**  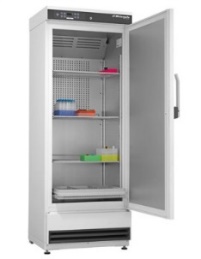 | **Subsequent processing or storage in the laboratory**   - Place samples in the refrigerator after sampling. - After collection, send samples to the Institute of Medical Psychology or initially store samples in your own laboratory freezer (-20°C, if available) until all samples can be sent collectively |

Instruction: *„You will find a straw and a little plastic container in the bag that we are about to hand over to you. Using the straw, pour the saliva into the container until a little more than half of it is filled. After use, you can dispose of the straws.* [Say this part only the first time]

*Collect the saliva in the frontal area of the mouth and press it through the straw.*

*When doing this, try not to blow through the straw! When you have collected sufficient saliva, please close the little container carefully (until it “clicks”) and put the saliva sample back in the corresponding bag****.* Enough saliva has been sampled when the container is a little more than half full!** *You can occasionally check the current amount of saliva in the container and continue to salivate until the required amount is reached. Please make sure to avoid bubbles forming in the sample.*
