# Supplementary material for: Social Support as a Stress Buffer or Stress Amplifier and the Moderating Role of Implicit Motives: Protocol for a Randomized Study
Source: JMIR Res Protoc. 2022 Aug 9;11(8):e39509. doi: 10.2196/39509 (PMC9399871; doi:10.2196/39509)
Supplement: Multimedia Appendix 12 [file resprot_v11i8e39509_app12.pdf]

29. MAI 2019

Deutsche
Forschungsgemeinschaft

Geistes- und Sozialwissenschaften 2: Sozial- und Verhaltenswissenschaften

Kennedyallee 40
53175 Bonn

Dr. Tanja Kollei

Telefon: +49 228 885-2321
Telefax: +49 228 885-713320
tanja.kollei@dfg.de

Fragen beantwortet:
Heike Kuhn

Telefon: +49 228 885-2593
Telefax: +49 228 885-713320
heike.kuhn@dfg.de
www.dfg.de

Deutsche Forschungsgemeinschaft · 53170 Bonn

Frau
Professorin Dr. Julia Schüler
Universität Konstanz
Geisteswissenschaftliche Sektion
Fach Sportwissenschaft
Postfach 30
78457 Konstanz

GZ: SCHU 2902/2-1 AOBJ: 658939 23.05.2019 GöN

Sehr geehrte Frau Professorin Schüler,

die Deutsche Forschungsgemeinschaft bewilligt Ihnen und Ihrer Hochschule entsprechend Ihrem Antrag, den Sie gemeinsam mit Frau Professorin Dr. Beate Ditzen zum Thema "Soziale Unterstützung als Stresspuffer oder Stressverstärker - Die moderierende Rolle sozialer Motive" gestellt haben, Mittel bis zur Höhe von 105.002 Euro zuzüglich 23.100 Euro Programmpauschale für 24 Monate.

Die Mittel werden als Drittmittelbewilligung zur Verfügung gestellt. Die Abrechnung erfolgt im Drittmittelverfahren. Es handelt sich um eine flexibilisierte Förderung im Sinne der Ziffer 7 der Verwendungsrichtlinien.

Im Einzelnen werden Ihnen für die Module - Basismodul - die folgenden Mittel bewilligt:

| | Anz. | Vol. | Dauer | Euro |
|---|---|---|---|---|
| **SCHU 2902/2-1** | | | | |
| **Professorin Dr. Julia Schüler** | | | **24 Mon.** | |
| durch DFG finanziert | | | | **128.102** |
| **Personalmittel** | | | | **93.502** |
| Postdoktorandin/Postdoktorand und Vergleichbare | 1 | 50% | 24 Mon. | 73.400 |
| Hilfskräfte | | | | 20.102 |

DFG

| | | | | |
|---|---|---|---|---|
| **Sachmittel** | | | | **11.500** |
| **Investitionsmittel** | | | | **-** |
| **Programmpauschale** | | | | **23.100** |

Dem darüber hinausgehenden Antrag konnte leider nicht entsprochen werden.

Sofern im Rahmen des Forschungsvorhabens Auslandsreisen durchgeführt werden, so sind die Sicherheitshinweise und Reisewarnungen des Auswärtigen Amtes zu berücksichtigen. Für Risiken, die sich aus einem Auslandsaufenthalt ergeben, kann die DFG keine Verantwortung übernehmen. Die DFG weist darauf hin, dass auch für Reisen im Rahmen dieser Bewilligung die allgemeinen Regelungen zur Genehmigung durch den Arbeitgeber bzw. Dienstherrn gelten.

Die bewilligten Sachmittel enthalten Publikationsmittel in Höhe von 1.500 Euro. Diese stehen Ihnen für die Publikation der wissenschaftlichen Ergebnisse ausschließlich dieses Projektes zur Verfügung. Sie können bis zu zwei Jahre nach Projektende im Bereich Finanzielle Umsetzung von Förderentscheidungen, E-Mail FIN2@dfg.de abgerufen werden.

Hinsichtlich der Programmpauschale beachten Sie bitte Ziffer 3.6 der Verwendungsrichtlinien (DFG-Vordruck 2.00 – 11/18).

Die DFG geht davon aus, dass bei der Planung und Durchführung von Forschung an Menschen, an identifizierbarem menschlichen Material und an identifizierbaren Daten die vom Weltärztebund (WMA - World Medical Association) im Juni 1964 verabschiedete Deklaration von Helsinki (Originaltitel: DECLARATION OF HELSINKI -Ethical Principles for Medical Research Involving Human Subjects) in der jeweils gültigen Fassung beachtet wird.

Die Stellungnahmen zu Ihrem Antrag liegen in anonymisierter Form bei.

Bei Fragen zur finanziellen Abwicklung der bewilligten Mittel wenden Sie sich bitte unter Angabe des Geschäftszeichens SCHU 2902/2-1 und des dazugehörigen Abrechnungsobjektes 658939 an den Bereich Finanzielle Umsetzung von Förderentscheidungen, E-Mail FIN2@dfg.de.

Die beigefügten Verwendungsrichtlinien (DFG-Vordruck 2.00 – 11/18) sind Bestandteil dieser Bewilligung.

Ihre Hochschule wird mit einem Schreiben gleichen Datums zum obigen Geschäftszeichen ebenfalls über den Umfang der Bewilligung informiert.

Sie werden gebeten, den Vertrauensdozenten Ihrer Hochschule für Angelegenheiten der Deutschen Forschungsgemeinschaft Herrn Professor Dr.

Giovanni Galizia, Postfach 623, 78457 Konstanz, von dieser Bewilligung zu unterrichten.

Frau Professorin Dr. Beate Ditzen (Geschäftszeichen DI 1716/4-1) wird mit einem gesonderten Schreiben über die ihren Antragsteil betreffende Entscheidung informiert.

Mit Annahme dieser Bewilligung verpflichten Sie sich, gleich nach Abschluss Ihres Projekts über die Ergebnisse zu berichten (siehe "Leitfaden für Abschlussberichte" in den beigefügten Verwendungsrichtlinien), wir haben dafür als Termin vorläufig den 01.09.2021 notiert.
Wenn Sie jedoch einen Fortsetzungsantrag zu diesem Projekt stellen, so berichten Sie bitte nur darin unter „Eigene Vorarbeiten" über Ihre bisherige Arbeit.

Projektergebnisse, die aus mit DFG-Mitteln finanzierten Projekten resultieren, müssen in geeigneter Art und Weise der Allgemeinheit zugänglich gemacht werden. Die Veröffentlichungen müssen einen Hinweis auf die DFG-Förderung enthalten. Hierbei sind ausschließlich die in Ziffer 13.1 der Verwendungsrichtlinien genannten Schreibweisen zu verwenden.

Ihre Projektnummer lautet 425213083.

Die zur Bearbeitung Ihres Antrags erforderlichen Daten werden von der DFG elektronisch gespeichert und verarbeitet.

Zu der hier bewilligten Fördermaßnahme werden personen- und institutionsspezifische Adress- und Kommunikationsdaten zur Person (Name, Institution und Ort, Telefon, Fax, E-Mail, www-Homepage) sowie inhaltserschließende Angaben (z. B. Thema, Zusammenfassung, Schlagwörter, fachliche Zuordnung, DFG-Verfahren, Förderzeitraum, Auslandsbezug) in dem Informationssystem GEPRIS (vgl.: http://www.dfg.de/gepris/) veröffentlicht. Zudem können diese Daten in anderen in Zusammenarbeit mit der DFG erstellten, nicht kommerziellen Publikationen und Datenbanken veröffentlicht werden.

Die Einwilligung zur Veröffentlichung kann – auch teilweise – jederzeit widerrufen werden, ohne dass dies die Rechtmäßigkeit der bis zum Widerruf erfolgten Verarbeitung berührt.

Die Deutsche Forschungsgemeinschaft wünscht Ihnen für Ihre Arbeit guten Erfolg.

Mit freundlichen Grüßen

Dr. Tanja Kollei

DFG

Stellungnahmen zum Antrag

Projekt: "Soziale Unterstützung als Stresspuffer oder Stressverstärker - Die moderierende Rolle sozialer Motive" (DI 1716/4-1 | SCHU 2902/2-1)

Antragstellende Personen: Frau Professorin Dr. Beate Ditzen, Heidelberg und Frau Professorin Dr. Julia Schüler, Konstanz

Gutachten 1:

"1. Wie beurteilen Sie die Qualität des Vorhabens, vor allem hinsichtlich Originalität und erwartetem Erkenntnisgewinn?

Das beschriebene Forschungsvorhaben nimmt eine innovative und für mehrere psychologische Disziplinen relevante Fragestellung in den Blick: Ist der Einfluss sozialer Unterstützung auf die subjektive und die physiologische Stressreaktion abhängig von der individuellen Ausprägung impliziter Motive? Die Kernhypothesen, dass affiliationsmotivierte Personen stärker von sozialer Unterstützung profitieren und machtmotivierte Personen entgegengesetzte Reaktionen zeigen sollten, sind konsistent mit der einschlägigen Forschungsliteratur und knüpfen gut an die bisherigen Forschungstätigkeiten der beiden Antragstellerinnen an. Ihre Untersuchung verspricht Einblicke in motivationale Mechanismen des bekannten differenziellen Effekts der sozialen Unterstützung auf Stressreaktionen, die sowohl für die psychologische Grundlagenforschung (insbes. Persönlichkeits- und Motivationspsychologie) als auch perspektivisch für verschiedene Anwendungsdisziplinen (Klinische, Pädagogische, Sport- und Arbeitspsychologie) interessant sind, z.B. für die Entwicklung von Stresspräventionsprogrammen unter Berücksichtigung motivationaler Persönlichkeitsunterschiede.

2. Inwiefern überzeugen Ziele und Arbeitsprogramm hinsichtlich der Klarheit der Arbeitshypothesen und einer sinnvollen Eingrenzung der Thematik? Benennen Sie bitte Stärken und Schwächen der geplanten Untersuchungen, die Angemessenheit der Methoden und des Zeitplans.

Die beschriebenen zentralen Methoden, TSST als experimentelles Paradigma und PSE zur Messung impliziter Motive, sind angemessen für die Untersuchung der genannten Forschungsfragen. Konkret schlagen die Antragstellerinnen zwei Studien vor. Studie 1 soll die erwarteten Interaktionseffekte zwischen experimentell manipulierter sozialer Unterstützung und individueller Motivausprägung testen. Darauf aufbauend sollen in Studie 2 spezifischere Formen der sozialen Unterstützung in einem sportpsychologischen Kontext untersucht werden. Da Studie 2 nur sinnvoll erscheint, sofern die Kernhypothesen in Studie 1 bestätigt werden können, schlage ich vor, die Förderung des Forschungsvorhabens zunächst auf die Durchführung von Studie 1 mit einer ausreichenden Teststärke zu konzentrieren. Dabei ist zu beachten, dass die bisherige Stichprobenplanung (Poweranalyse) nur für eine der zwei vorgesehenen experimentellen Gruppen gültig ist. Um eine akzeptable Teststärke für die Prüfung der zentralen Moderationshypothesen zu erhalten, sollte der vorgeschlagene Stichprobenumfang (N = 154) daher mindestens verdoppelt werden. Eine solche Studie wäre innerhalb eines Förderungszeitraums von zwei Jahren mit überschaubaren Änderungen der beantragten Mittel im Bereich der studentischen Hilfskräfte sowie der Probandenhonorare und Materialkosten umzusetzen. Bei erfolgreicher Bestätigung der zentralen Hypothesen in einer so erweiterten

Studie 1, könnten im Anschluss spezifischere und anwendungsbezogene Fragestellungen im Rahmen von Folgeprojekten ins Auge gefasst werden.

3. Wie bewerten Sie die Tragfähigkeit der Vorarbeiten und die Qualität der Veröffentlichungen (siehe hierzu die Hinweise zu Publikationsverzeichnissen) und die Qualifikation der / des Antragstellenden – allgemein und hinsichtlich des konkreten Projekts? Ist er/sie qualifiziert, das beantragte Projekt selbstverantwortlich zu leiten?
Beide Antragstellerinnen haben bereits in der Vergangenheit erfolgreich Drittmittelprojekte geleitet und zeichnen sich durch langjährige hochrangige Publikationstätigkeit aus. Besonders relevant sind hier Prof. Schülers Forschung zu impliziten Motiven (insbes. als Moderatoren psychobiologischer Prozesse) und Prof. Ditzens Arbeiten im Bereich der psychobiologischen Forschung zu Stress in sozialen Kontexten. Da mit der Anwendung und Auswertung impliziter Motivtests sowie mit der hormonbezogenen Analyse von Speichelproben zwei recht spezifische methodische Kompetenzen für das Gelingen des Forschungsvorhabens erforderlich sind, ergänzen sich die Expertisen der beiden Antragstellerinnen hervorragend. Hinzu kommt die Kollaboration mit Prof. Oliver Schultheiss, der Experte auf beiden Gebieten ist. Insgesamt habe ich daher keine Zweifel, dass das Forschungsvorhaben von den Antragstellerinnen erfolgreich durchgeführt werden kann.

4. Wie schätzen Sie Arbeitsmöglichkeiten und das wissenschaftliche Umfeld in der Einrichtung ein, in der das Projekt durchgeführt werden soll?
Die notwendigen Rahmenbedingungen (Forschungsinfrastruktur) sind an beiden Standorten gegeben; insbesondere ist die kompetente Analyse der Speichelproben durch das biochemische Labor des Zentrums für Psychosoziale Medizin in Heidelberg, dem Prof. Ditzen vorsteht, gewährleistet.

5. Bitte formulieren Sie ein eindeutiges Votum für oder gegen eine Förderung. Bitte machen Sie bei einem Votum für eine Förderung einen konkret ausdifferenzierten Mittelvorschlag. Berücksichtigen Sie dabei gegebenenfalls, ob die beantragten Mittel angemessen sind.
Unter Berücksichtigung der oben genannten Änderungsvorschläge befürworte ich die Förderung des Forschungsvorhabens ausdrücklich. Konkret beziehen sich die vorgeschlagenen Änderungen auf (1) den Wegfall von Studie 2, (2) eine Verkürzung des Förderungszeitraums auf zwei Jahre, (3) eine Vergrößerung der Stichprobe um mind. 100%. Das durch (3) erhöhte Arbeitsvolumen (im Vergleich zur aktuellen Planung von Studie 1) in den Bereichen Rekrutierung, Datenerhebung und –auswertung lässt sich durch eine Verdopplung der für Studie 1 beantragten Mittel für Probandenhonorare, eine Erhöhung der beantragten Arbeitsstunden für studentische Hilfskräfte um jeweils 50% (d.h. von 40 auf 60 Monatsstunden am Standort Konstanz und von 20 auf 30 Monatsstunden am Standort Heidelberg) sowie durch die Verdopplung der aktuell für Studie 1 beantragten Sachmittel (Material) für die Hormonanalysen am Standort Heidelberg kompensieren. Da die Kosten für Studie 2 entfallen und alle anderen beantragten Mittel (Post-Doc-Stelle, Reisemittel etc.) aufgrund der Verkürzung des Förderungszeitraums um ein Drittel reduziert werden können, entstehen dadurch keine Mehrkosten, sondern Einsparungen gegenüber den aktuell beantragten Fördermitteln. Ich möchte betonen, dass diese Änderungsvorschläge nicht als Kritik am Forschungsvorhaben an sich zu verstehen sind. Vielmehr sollen die Erfolgschancen des Projekts sowie der effiziente Einsatz der Fördermittel dadurch optimiert werden."

Gutachten 2:

"1. Qualität des Vorhabens (Originalität und erwarteter Erkenntnisgewinn)
Im theoretischen Teil begründen die Antragsteller auf überzeugende Weise die wissenschaftliche Relevanz ihrer Fragestellung, indem dargelegt wird, dass ein Erklärungsansatz für konfligierende Ergebnisse bezüglich der Wirkung von sozialer Unterstützung, in der Vernachlässigung des Einflusses der individuellen Ausprägung sozialer Motive liegen könnte. Der Antrag ist sehr stringent und gut lesbar. Die Erforschung der genauen Wirkmechanismen von Resilienzfaktoren, wie soziale Unterstützung, welche das Potential haben, die negativen Auswirkungen von Stressbelastung zu reduzieren, liefert zum einen die theoretischen Grundlagen für die Entwicklung und Bewertung von Präventionsansätzen. Zum anderen adressieren die Antragsteller mit der Betrachtung individueller impliziter Motivlagen sowie möglicher Geschlechtseffekte eine bedeutsame Lücke in der gegenwärtigen Stress-und Präventionsforschung, in der interindividuelle Unterschiede noch oft vernachlässigt werden und „One Size Fits All"-Ansätze weiterhin dominieren. Zusammenfassend ist der vorgeschlagene Fokus auf den Einfluss der individuellen Ausprägung von implizitem Bindungs- und Machtmotiv auf die Wahrnehmung von sozialer Unterstützung und deren Auswirkungen auf psychobiologische Stressreaktionen hoch originell und von direkter sozialer Relevanz für den Anwendungskontext.

2. Ziele und Arbeitsprogramm (Klarheit der Arbeitshypothesen, Stärken und Schwächen der geplanten Untersuchungen, Angemessenheit der Methoden und des Zeitplans)

Die Ziele und das Arbeitsprogramm des Antrags sind klar konzeptualisiert und die Arbeitshypothesen leiten sich schlüssig aus den theoretischen Grundlagen und empirischen Vorbefunden ab. Studie 1 adressiert zunächst die Frage, ob soziale Unterstützung motiv-spezifische Effekte auf die psychobiologische Stressreaktion ausübt und ob ein motiv-spezifisches Arousal mit motiv-spezifischen Geschlechtshormonreaktionen einhergeht. Hier werden auch geschlechtspezifische Interaktionseffekte postuliert. Studie 2 untersucht dann in einem zweiten Schritt in einer rein männlichen Stichprobe, ob Individuen mit einem stark ausgeprägten Bindungsmotiv stärker von emotionaler versus informationsbezogener sozialer Unterstützung profitieren. Die vorgeschlagenen experimentellen Forschungsmethoden (Induktion einer akuten Stresssituation mittels TSST, Erfassung der Stressreaktion über a) die physiologischen Parameter Cortisol und SAA und damit Integration von HHNA und SNS sowie b) über subjektive Stressratings, Erfassung des motiv-spezifischen Affekts über eine Adjektiv-Liste sowie motiv-spezifischen Reaktionen der Sexualhormone Progesteron und Testosteron auf soziale Unterstützung) sind geeignet um die Forschungsfrage von Studie 1 zu beantworten. Relevante Kontrollvariablen werden berücksichtigt. Etwas unklar bleibt lediglich, wann die Messung der impliziten sozialen Motive erfolgt. In Abbildung 2 scheint es, als würde sie nach der Einteilung in die experimentellen Gruppen im Rahmen der Labortestung erfolgen. Im Abschnitt „Participants" heisst es: „First, participants will fill in a websurvey that contains information about the study, the informed consent form and the implicit motive measure." Kann davon ausgegangen werden, dass sich Bindungs- und Machtmotiv etwa gleich auf die Stichprobe verteilen? Auch wird nicht angegeben, ob die Teilnehmer von Studie 1 in einer bestimmten Altersspanne rekrutiert werden sollen. In vorherigen Studien konnten zum Teil Alterseffekte auf die Ausprägung impliziter Motive

nachgewiesen werden. Allerdings ist die Befundlage hier auch nicht eindeutig. Auch das Forschungsdesign von Studie 2 (insbesondere die Operationalisierung von informationsbezogener und emotionaler sozialer Unterstützung) wird schlüssig dargelegt. Lediglich die Argumentation, warum eine Adaptation des TSST in Studie 2 zur Beantwortung der Forschungsfrage sinnvoll ist, fällt etwas knapp aus. Der Zeitplan ist ehrgeizig, aber vor dem Hintergrund der Expertise und Vorerfahrung des Teams realistisch.

3. Qualifikation der / des Antragstellenden (allgemein und hinsichtlich des konkreten Projekts)
Beide Antragstellerinnen haben auf hohem Niveau zu dem übergeordneten Antragsthema (Schwerpunkt Ditzen: Psychobiologische Stressforschung sowie Soziale Unterstützung; Schwerpunkt Schüler: Implizite Motive) publiziert und verfügen über die notwendige fachliche Expertise. Die Unterstützung des Projekts durch den vorgeschlagenen Kollaborationspartner Oliver Schultheiss erscheint vor dem Hintergrund seiner Expertise, insbesondere im Bereich Hormonreaktionen im Zusammenhang mit impliziten Motiven, sinnvoll und vielversprechend.

4. Arbeitsmöglichkeiten und das wissenschaftliche Umfeld
Das wissenschaftliche Umfeld der Universitäten Konstanz und Heidelberg ist hervorragend dazu geeignet das Projekt im vorgeschlagenen Zeitplan umzusetzen.

5. Abschließendes Votum
Ich halte das Gesamtkonzept des Antrags für Erfolg versprechend und befürworte eine Förderung. Die beantragten Projektmittel sind realistisch und nachvollziehbar begründet."

<u>Stellungnahme des Fachkollegiums:</u>
"Beide Gutachten bescheinigen diesem Vorhaben hohe Originalität, eine überzeugende theoretische Herleitung der Fragestellung, und eine angemessene methodische Umsetzung und empfehlen die Förderung des Antrags. Das Fachkollegium schließt sich dieser positiven Einschätzung an, der Hinweis von Gutachten I bezüglich der zu geringen Power in Studie ist aber ebenfalls sehr nachvollziehbar. Daher wird der Antrag zur Förderung empfohlen, aber mit einer verkürzten Laufzeit von 24 Monaten und der Empfehlung, sich auf Studie 1 zu fokussieren und bei dieser die Stichprobe deutlich zu erhöhen."
